# Profiling miRNA changes in Epstein-Barr virus lytic infection identifies a function for BZLF1 in upregulating miRNAs from the DLK1-DIO3 locus

**DOI:** 10.1371/journal.ppat.1013347

**Published:** 2025-07-17

**Authors:** Ashley M. Campbell, Victoria C. Taylor, Beata Cohan, Lori Frappier

**Affiliations:** Department of Molecular Genetics, University of Toronto, Toronto, Canada; University of North Carolina at Chapel Hill Medical School, UNITED STATES OF AMERICA

## Abstract

Cellular and viral miRNAs are thought to play important roles in regulating Epstein-Barr virus (EBV) latent and lytic infections, however, to date, most studies have focussed on latent infections in B cells. To determine how cellular and viral miRNAs contribute to EBV lytic infection in epithelial cells, the main sites of lytic infection, we conducted miRNA-sequencing experiments in EBV-infected AGS gastric carcinoma cells, before and after reactivation to the lytic cycle, analysing both total miRNA and Ago2-associated miRNAs. We identified over 100 miRNAs whose association with Ago2 was affected upon EBV reactivation, most of which were due to changes in miRNA abundance. For EBV miRNAs, the most striking result was that the BHRF1 miRNAs, previously only reported to be expressed in B cells, were upregulated upon reactivation. The largest changes in cellular miRNAs upon EBV reactivation were increases in the abundance and Ago2-association of miR-409-3p, miR-381-3p and miR-370-3p, which appear to have pro-viral effects. In particular, inhibiting miR-409-3p reduced BZLF1 and other EBV lytic protein expression, at least in part through modulation of ZEB1. Interestingly, these miRNAs all originate from the DLK1-DIO3 locus (14q32.2 - 32.31), which encodes multiple lncRNAs. We showed that the lncRNAs *MEG9, MIR381HG, and MEG8*, from which miR-409-3p, miR-381-3p and miR-370-3p are derived, were also upregulated upon reactivation in AGS and nasopharyngeal carcinoma cells lines and occurred very early in the lytic cycle at the time of BZLF1 expression. In keeping with this timing, BZLF1 was sufficient to induce these lncRNAs dependent on its transactivation activity, and was detected at a key DLK1-DIO3 control element, consistent with a direct role in transcriptional activation. Therefore, we have identified a new role for BZFL1 in activating the expression of lncRNAs in the DLK1-DIO3 locus, resulting in induction of a subset of encoded miRNAs that promote lytic infection.

## Author summary

Epstein-Barr virus (EBV) infects most people worldwide, contributing to the development of multiple sclerosis and several types of cancer, including gastric and nasopharyngeal carcinoma. While miRNAs are important regulators of viral and cellular gene expression, there is little known about how cellular and EBV miRNAs contribute to and regulate EBV lytic infection. Here we profile changes in miRNAs that occur upon reactivation of EBV from latent to lytic infection, and identify cellular miRNAs from the DLK1-DIO3 locus in chromosome 14 that are dramatically upregulated in EBV lytic infection and promote the lytic cycle. We further show that the induction of these miRNAs results from the action of the EBV lytic switch protein, which associates with this locus and induces the expression of the precursor RNAs from which the miRNAs are derived. Our study provides new insights into how cellular miRNAs can be used to promote EBV infection and a new role for an EBV protein in dysregulating cellular miRNA expression.

## Introduction

miRNAs are evolutionarily conserved, small non-coding RNAs (21-25 nucleotides) important for mRNA regulation [1,2]. miRNAs are considered global regulators of the transcriptome as the >1800 annotated human miRNAs (hsa-miRNAs) can target >60% of human protein-coding transcripts [3]. Hsa-miRNAs are encoded within the genome as individual miRNAs or as polycistronic transcripts called clusters [4,5]. The latter are chromosomal regions varying in length up to ~100 kb and can contain several miRNAs [6]. The largest clusters within the human genome include the Chromosome 19 miRNA Cluster (C19MC) and Chromosome 14 miRNA Cluster (C14MC), encoding 46 [7,8] and 52 [9] miRNAs, respectively.

Small noncoding RNAs, including miRNAs, have also been identified in several human viruses, including adenoviruses [10,11], orthoebolaviruses [12], hepatitis B virus, polyomaviruses [13], and herpesviruses [14]. Epstein-Barr virus (EBV), a gamma-herpesvirus, was the first virus shown to encode miRNAs [15]. These miRNAs are organized in the BHRF1, BART1 and BART2 clusters. The BHRF1 miRNAs (miR-BHRF1) flank the BHRF1 gene while most of the BART1 and BART2 clusters lay within a ~12 kbp region that is deleted in the common lab strain, B95.8 [16]. Complete EBV genomes encode 25 pre-miRNAs that are processed to 44 mature miRNAs. Four miRNAs originate from the BHRF1 cluster, while the remaining 40 are located within the BART clusters. The expression profile of these miRNAs is linked to latency type, where BART miRNAs are expressed in all latency types, but expression of BHRF1s miRNAs is restricted to latency III [16].

Several roles have been identified for EBV miRNAs, including promoting latent and lytic infections, immune evasion, and preventing apoptosis [3,15]. While the functions of many EBV miRNAs are not well characterized, some EBV miRNAs have been shown to target viral transcripts to regulate EBV lytic infection [17,18] and to target cellular transcripts to modulate the cellular environment [19–28]. There is also evidence that cellular miRNAs play important roles in regulating various stages of EBV infection. For example, upon EBV infection of primary B cells, hsa-miR-155 is induced by EBNA2, which appears to contribute to EBV immortalization by modulating NF-κB signalling [29]. Other cellular miRNAs, including miR-200b and miR-429, have been shown to be inversely correlated with ZEB1 and ZEB2/SIP1, which are key regulators of BZLF1 gene expression [30–32]. In addition, the expression of miR-200b and miR-429 is downregulated upon infection of primary B cells, while upregulated in plasma cell lines and upon BCR activation, both of which promote EBV reactivation [33,34], suggesting a role for these miRNAs in inducing lytic infection.

While there have been several miRNA-seq experiments examining changes in miRNAs in latent EBV infections [35–43], comprehensive analyses of changes in cellular and viral miRNA expression in EBV lytic infection are lacking. To gain a better understanding of what viral and cellular miRNAs may contribute to EBV lytic infection, we conducted Ago2-immunoprecipitation followed by miRNA-seq in AGS cells latently infected with EBV, before and after reactivation to the lytic cycle. While most EBV miRNAs increased in expression and Ago2-association in lytic infection to some degree, two BHRF1 miRNAs (miR-BHRF1-2-3p and BHRF1-3) increased dramatically upon reactivation of multiple EBV-positive epithelial cell lines. The biggest changes in cellular miRNAs upon EBV reactivation were increases in multiple miRNAs from C14MC within the DLK1-DIO3 locus; changes that were consistent in multiple EBV-infected epithelial cell lines. We further show that transcripts giving rise to these miRNAs are similarly increased in lytic infection and that the EBV immediate early transactivator, BZLF1, was sufficient to induce these transcripts.

## Results

### Analysis of miRNA usage in EBV latent and lytic infection

While miRNAs are known to play important roles in EBV latent infections, little is known about miRNA usage in EBV lytic infection and how miRNA profiles are altered upon reactivation from latent to lytic infection. To address these questions, we required cells latently infected with EBV that could be efficiently reactivated to the lytic cycle. We took advantage of AGS-EBV-Z cells that we previously showed reactivate efficiently upon doxycycline (dox) treatment due to the presence of a dox-inducible lytic switch protein, BZLF1 [44]. In quadruplicate experiments, lysates of AGS-EBV-Z cells were generated from cells treated with dox for 48 hrs (lytic) or left untreated (latent). A sample of lysate was kept as an input sample, while the remaining lysate was used for Ago2-immunoprecipation to capture miRNAs loaded into Ago2. The Ago2-bound and input miRNAs were then isolated, and small RNA cDNA libraries were generated for sequencing. Sequencing resulted in a minimal read depth of 5 million reads (S1 Fig). miRNAs with less than 10 reads per million and that were not identified in all eight samples were excluded from the analysis. In total, 342 miRNAs were identified across all samples that were further analyzed (S1 Table).

**Fig 1:**
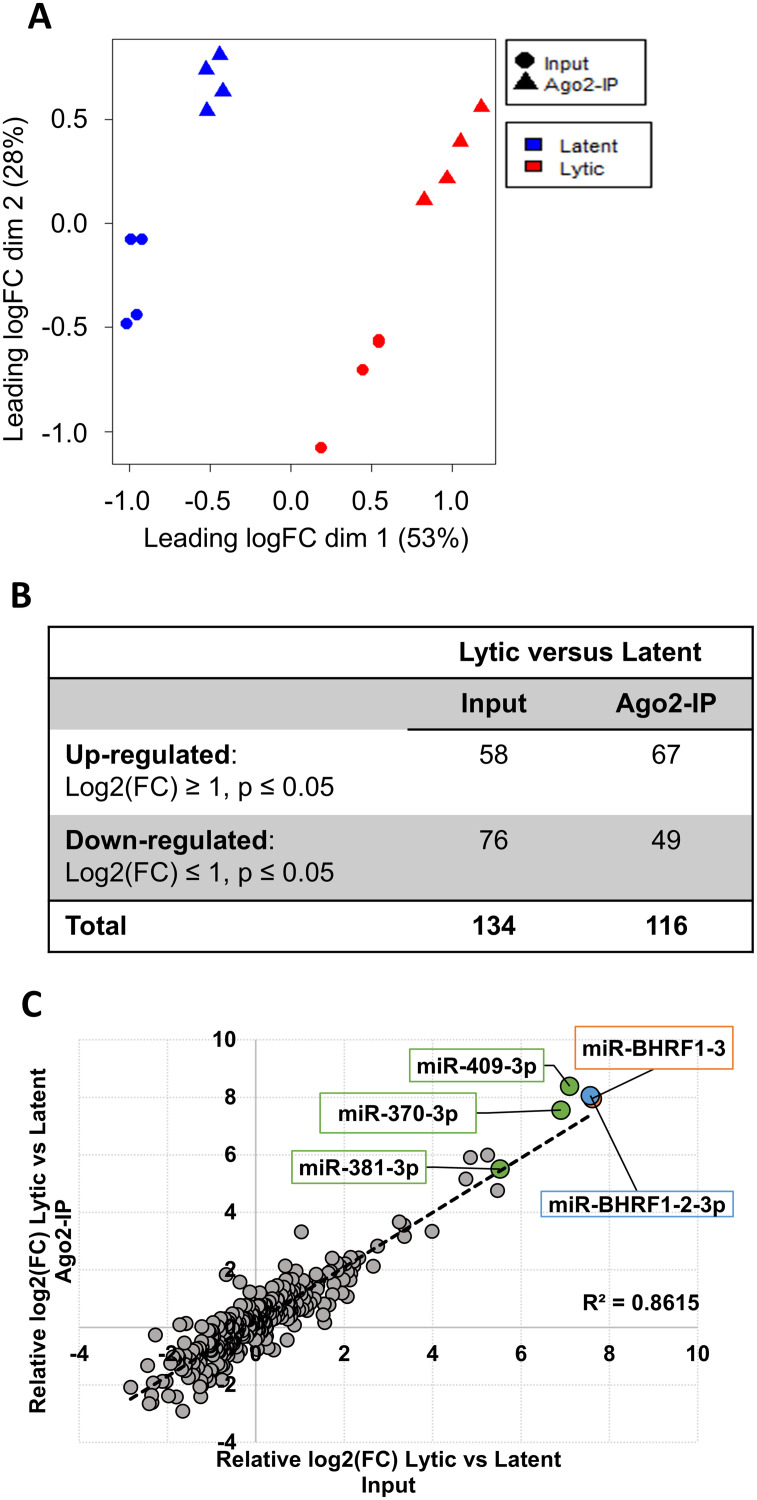
Analysis of miRNA libraries from latent and lytic AGS-EBV-Z cells. **(A)** MDA plot of input (circles) and Ago2-IP (triangle) samples from EBV latent (blue) and lytic (red) infections. **(B)** Table summarizing miRNAs significantly (p ≤ 0.05) and differentially expressed |Log2(FC)| ≥ 1 for lytic versus latent infection. **(C)** Scatterplot of miRNA differential expression with lytic versus latent input (x-axis) and Ago2-IP (Y-axis) samples. The top differentially expressed miRNAs are indicated, with EBV miR-BHRF1-3 and miR-BHRF1-2-3p in orange and blue, respectively, and cellular miRNAs, miR-409-3p, miR-381-3p, and miR-370-3p marked in green.

Multidimensional scaling (MDS) analysis of the 342 miRNAs showed that replicates of total or Ago2-associated miRNAs from the same infection stage clustered together and that the most variation was between latent and lytic conditions, with input and Ago2-IP samples within one infection mode being more similar to each other (Fig 1A). Of the 342 miRNAs analyzed, 134 miRNAs were found to be significantly (p ≤ 0.05) differentially expressed (fold change ≥ |2|) in the input samples upon reactivation, with 58 increased and 76 decreased (Fig 1B). There was a linear relationship between the differentially expressed miRNAs in the input and Ago2-IP samples (Fig 1C), suggesting most changes in Ago2-associated miRNAs are due to changes in miRNA abundance. However, 116 miRNAs were found to be significantly differentially expressed in the Ago2-IPs, suggesting that their incorporation into Ago2 is not solely dependent on their cellular levels (see S1 Table).

### Changes in EBV miRNAs in latent and lytic infection

**Fig 2:**
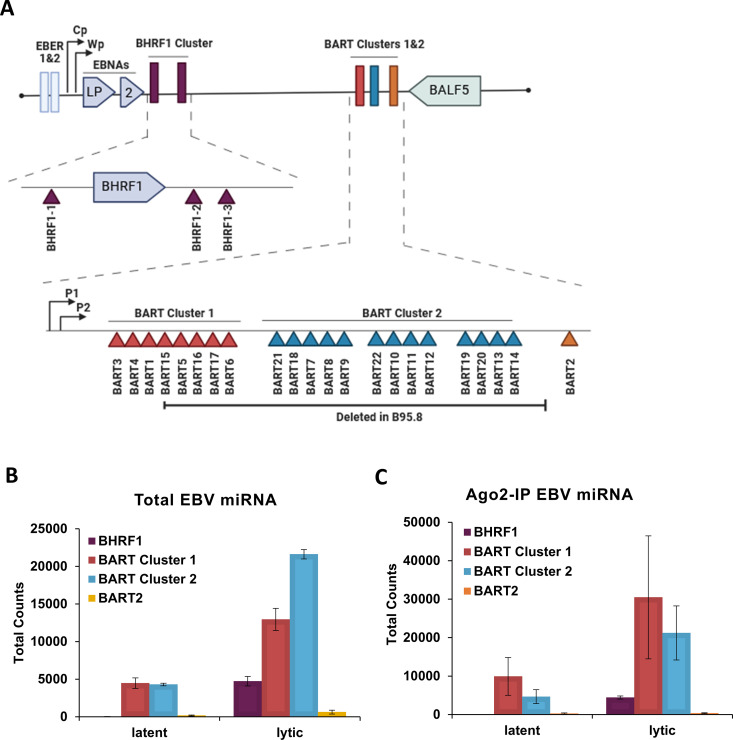
EBV-derived miRNAs increase in abundance upon lytic infection. **(A)** Schematic of EBV miRNA clusters and BART2 miRNA within the EBV genome. **(B)** Quantification of total EBV miRNAs, from miR-BART2 or the indicated miRNA clusters, in latent and lytic infection. **(C)** Quantification of EBV miRNAs from Ago2-IPs like the input lysates in **B.**

The RNA-seq experiments identified 31/44 EBV-derived miRNAs in the input and Ago2-IP samples (Table 1). In latent infection, EBV miRNAs accounted for 0.76 ± 0.22% of total miRNAs in input samples, and this increased to 4.43 ± 0.54% in lytic infection. The EBV miRNAs were derived from all known clusters in the EBV genome (BHRF1 cluster and BART clusters 1 and 2; Fig 2A). The level of EBV miRNAs from all clusters increased in lytic infection in both total abundance and Ago2-association (Fig 2B and 2C). Of the miRNAs in the BART clusters, miR-BART7-5p stood out as the most highly induced in lytic infection. However, the biggest fold-change was in two miRNAs from the BHRF1 cluster, miR-BHRF1–2-3p and miR-BHRF1–3, which were increased 190- and 195-fold, respectively (Table 1). These two miRNAs were underrepresented (< 1%) in latent infections but accounted for ~12% of the EBV miRNAs in lytic infection, suggesting that they may play a role in EBV lytic infection.

**Table 1:** EBV miRNAs Differentially Expressed Upon EBV Reactivation.

	miRNA	Lytic versus Latent			
		Input		Ago2-IP	
		Fold change	PValue	Fold change	PValue
BHRF1 Cluster	ebv-miR-BHRF1–1	ND*		ND	
	ebv-miR-BHRF1–3	195.36	1.25E-44	249.00	1.31E-47
	ebv-miR-BHRF1–2-5p	ND		ND	
	ebv-miR-BHRF1–2-3p	190.02	4.60E-101	266.87	6.70E-110
BART Cluster 1	ebv-miR-BART3-5p	ND		ND	
	ebv-miR-BART3-3p	ND		ND	
	ebv-miR-BART4-5p	1.40	1.54E-01	1.59	4.93E-02
	ebv-miR-BART4-3p	1.72	3.25E-02	2.28	8.45E-04
	ebv-miR-BART1-5p	3.39	1.13E-05	2.85	1.57E-04
	ebv-miR-BART1-3p	2.95	3.60E-03	1.13	7.49E-01
	ebv-miR-BART15	ND		ND	
	ebv-miR-BART5-5p	ND		ND	
	ebv-miR-BART5-3p	ND		ND	
	ebv-miR-BART16	3.12	4.87E-05	3.71	3.29E-06
	ebv-miR-BART17-5p	3.76	4.86E-10	4.38	5.35E-12
	ebv-miR-BART17-3p	1.83	1.11E-02	1.06	8.17E-01
	ebv-miR-BART6-5p	1.73	3.40E-02	1.30	3.04E-01
	ebv-miR-BART6-3p	2.50	9.98E-06	1.99	8.67E-04
BART Cluster 2	ebv-miR-BART21-5p	ND		ND	
	ebv-miR-BART21-3p	ND		ND	
	ebv-miR-BART18-5p	2.19	2.26E-02	2.20	2.23E-02
	ebv-miR-BART18-3p	4.06	6.61E-10	4.63	1.69E-11
	ebv-miR-BART7-5p	44.32	2.54E-58	27.10	1.58E-47
	ebv-miR-BART7-3p	4.20	1.71E-08	2.10	2.63E-03
	ebv-miR-BART8-5p	3.58	6.18E-08	3.94	6.32E-09
	ebv-miR-BART8-3p	4.41	1.06E-14	3.66	1.09E-11
	ebv-miR-BART9-5p	10.27	6.22E-17	9.00	2.00E-15
	ebv-miR-BART9-3p	2.62	8.53E-06	2.51	1.85E-05
	ebv-miR-BART22	6.73	3.04E-16	7.11	5.53E-17
	ebv-miR-BART10-5p	ND		ND	
	ebv-miR-BART10-3p	4.20	1.24E-05	3.84	4.20E-05
	ebv-miR-BART11-5p	2.11	1.45E-04	1.97	5.30E-04
	ebv-miR-BART11-3p	2.20	1.38E-03	1.69	3.11E-02
	ebv-miR-BART12	3.56	3.42E-08	4.76	1.89E-11
	ebv-miR-BART19-5p	3.86	3.99E-05	2.28	1.08E-02
	ebv-miR-BART19-3p	4.66	6.65E-13	4.44	3.00E-12
	ebv-miR-BART20-5p	ND		ND	
	ebv-miR-BART20-3p	ND		ND	
	ebv-miR-BART13-5p	5.03	1.35E-10	5.35	2.96E-11
	ebv-miR-BART13-3p	2.20	3.69E-04	2.69	8.63E-06
	ebv-miR-BART14-5p	3.14	4.89E-05	1.72	4.97E-02
	ebv-miR-BART14-3p	3.23	1.12E-06	3.86	2.52E-08
	ebv-miR-BART2-5p	2.87	4.85E-04	1.35	3.11E-01
	ebv-miR-BART2-3p	ND		ND	

*ND: not detected/below threshold

**Fig 3:**
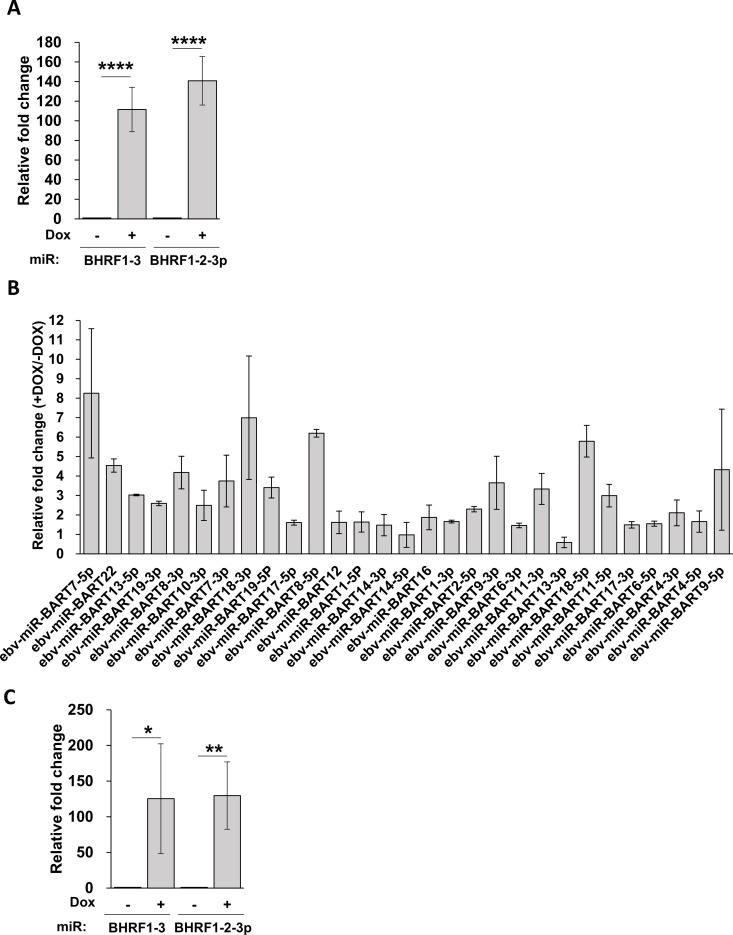
Validation of increases in EBV miRNAs. (A and B) miRNAs were extracted from AGS-EBV-Z cells before or after reactivation with dox, then miR-BHRF1–3 and miR-BHRF1–2-3p (A) or BART miRNAs (B) were quantified by RT-qPCR relative to miR-151a-3p. (C) miRNAs were extracted from NPC43-Z cells before or after reactivation with dox, then miR-BHRF1–3 and miR-BHRF1–2-3p were quantified as in A. * = 0.01 < P ≤ 0.05, ** = 0.001 < P ≤ 0.01, **** = P ≤ 0.0001.

The changes in EBV miRNAs upon lytic reactivation were validated in AGS-EBV-Z cells using RT-qPCR (Fig 3). The RNA-seq data showed a cellular miRNA, hsa-miR-151a-3p (miRbase: MI0000809), did not change between latent and lytic infection, and therefore, miR-151a-3p was used as a normalization control for miRNA analysis during EBV infection. The RT-qPCR data confirmed the miRNA seq data, including the finding that the top differentially expressed EBV miRNAs were miR-BHRF1–2-3p and miR-BHRF1–3 (Fig 3A and 3B). We also examined whether the increase in BHRF1 miRNAs in lytic infection was conserved in a patient-derived EBV-positive nasopharyngeal carcinoma cell line, NPC43 [45]. A version of NPC43 containing a dox-inducible BZLF1 cassette to facilitate efficient reactivation (NPC43-Z) was used for these experiments [44]. NPC43-Z showed a similar dramatic increase in miR-BHRF1–3 and miR-BHRF1–2-3p upon reactivation as in AGS-EBV-Z (Fig 3C), showing that this increase is not specific to AGS cells.

### miR-409-3p, miR-381-3p and miR-370-3p are highly induced upon EBV reactivation in multiple epithelial cell lines and promote lytic infection

**Fig 4:**
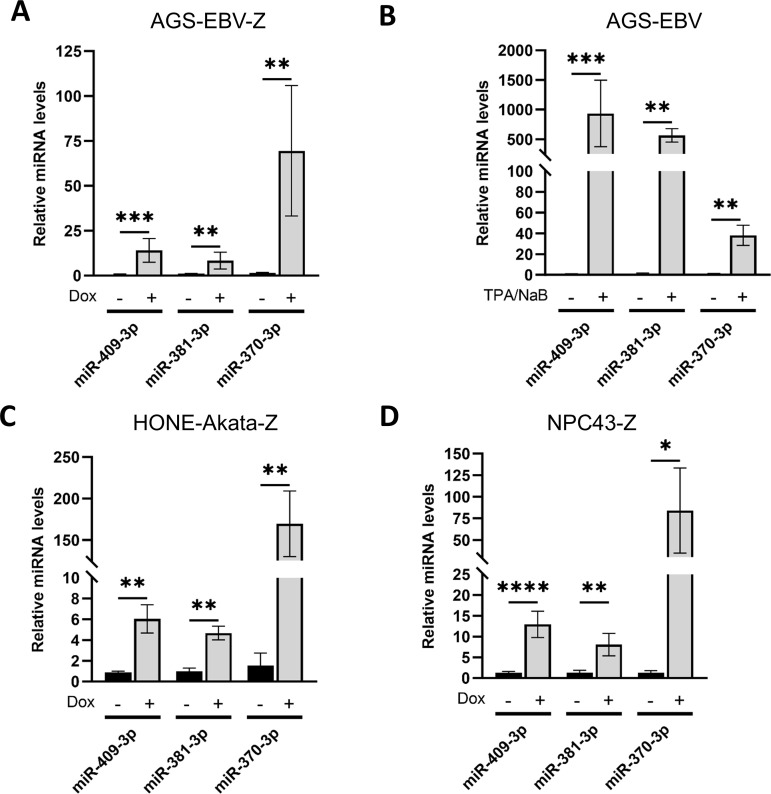
miR-409-3p, miR-381-3p and miR-370-3p increase upon reactivation. (A) miRNAs were extracted from AGS-EBV-Z cells before or after reactivation with dox, then miR-409-3p, miR-381-3p, and miR-370-3p were quantified by RT-qPCR and normalized to miR-151a-3p. (B) miRNAs were extracted from AGS-EBV cells before and after reactivation with TPA/NaB, then miRNAs were quantified as in A. The same experiment as A, except with HONE-Akata-Z (C) or NPC43-Z cells (D). * = 0.01 < P ≤ 0.05, ** = 0.001 < P ≤ 0.01, *** = 0.0001 < P ≤ 0.001, **** = P ≤ 0.0001.

The miRNA-seq analysis identified 31 cellular miRNAs that were significantly (p ≤ 0.05) upregulated and 76 cellular miRNAs that were significantly downregulated more than 2-fold upon EBV reactivation from latent to lytic infection (S1 Table). The top 10 cellular miRNAs that were increased or decreased are shown in Table 2. Of these, the biggest changes in miRNA levels were increases in miR-409-3p, miR-370-3p, and miR-381-3p. These miRNAs were lowly expressed, but detectable during latency, then increased to be in the top 10% of miRNAs during lytic infection. miR-409-3p and miR-370-3p levels were similar to let-7d-5p, which has previously been shown to target PRDM5 in gastric carcinoma cell lines, including AGS, to promote cell proliferation, migration, invasion and reduce apoptosis [46]. The increases in miR-409-3p, miR-370-3p, and miR-381-3p upon reactivation were validated in AGS-EBV-Z cells by RT-qPCR (Fig 4A). They were also validated in the parental AGS-EBV cells reactivated with TPA/NaB treatment (Fig 4B), showing that the induction of these miRNAs was not a result of the pTRIPZ inducible system in AGS-EBV-Z cells. We then determined whether this increase also occurs upon EBV lytic reactivation in other cell backgrounds. To this end, HONE-Akata-Z and NPC43-Z cell lines (both containing dox-inducible BZLF1) were reactivated to lytic infection with dox treatment, and miR-409-3p, miR-381-3p, and miR-370-3p were quantified by RT-qPCR. All three miRNAs were increased in lytic infection in both cell lines (Fig 4C and 4D), indicating that induction of miR-409-3p, miR-381-3p, and miR-370-3p occurs upon EBV reactivation in multiple epithelial cell lines.

**Table 2:** Top 10 Up- And Down-Regulated Cellular miRNAs Upon EBV Reactivation.

	Lytic versus Latent			
	Input		Ago2-IP	
miRNA	Fold change	P-value	Fold change	P-value
hsa-miR-409-3p	137.19	1.78E-28	335.46	2.79E-35
hsa-miR-370-3p	119.43	4.36E-16	187.40	5.19E-18
hsa-miR-381-3p	45.89	3.29E-28	45.25	3.97E-28
hsa-miR-375-3p	37.79	2.93E-49	63.56	2.20E-60
hsa-miR-143-3p	28.84	3.30E-17	60.13	1.74E-22
hsa-miR-139-5p	26.91	3.15E-25	35.75	1.59E-28
hsa-miR-212-5p	15.89	4.18E-25	10.13	6.43E-20
hsa-miR-514a-3p	10.20	1.54E-18	11.71	4.25E-20
hsa-miR-6842-3p	9.51	6.43E-11	12.73	2.89E-13
hsa-miR-215-5p	6.32	4.76E-13	4.38	5.12E-09
hsa-miR-378i	-3.92	2.55E-11	-5.21	6.23E-16
hsa-miR-130a-3p	-4.08	1.08E-08	-3.68	1.04E-07
hsa-miR-34a-5p	-4.35	1.82E-07	-3.68	5.10E-06
hsa-miR-25-5p	-4.82	9.63E-06	-1.20	6.00E-01
hsa-miR-302b-3p	-4.99	3.96E-09	-3.84	6.49E-07
hsa-miR-378d^†^	-5.10	1.22E-07	-6.15	3.84E-09
hsa-miR-17-5p	-5.13	9.17E-14	-5.17	7.80E-14
hsa-miR-378d^†^	-5.31	1.52E-07	-6.28	7.06E-09
hsa-miR-23b-5p	-5.46	3.06E-09	-2.51	0.000806
hsa-miR-107	-7.11	4.03E-19	-4.23	2.10E-11

†hsa-miR-378d was identified from two different precursor miRNAs.

**Fig 5:**
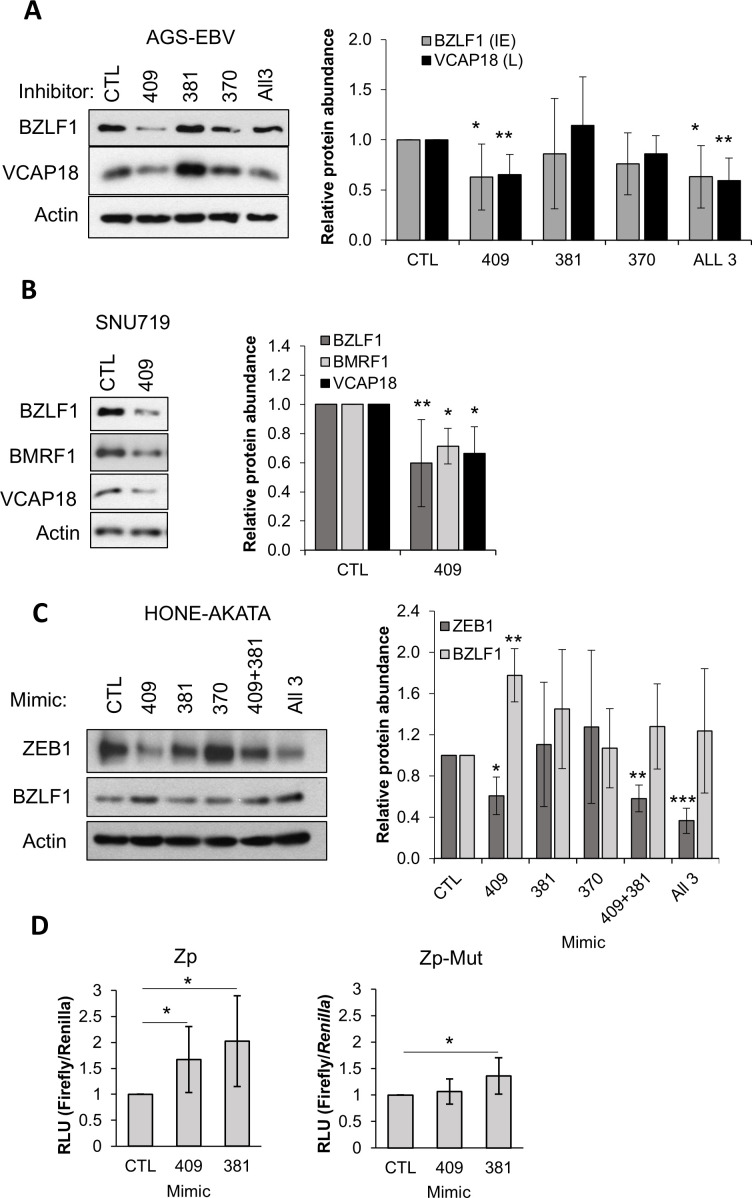
Effects of miR-409-3p, miR-381-3p and miR-370-3p on lytic infection and ZEB1. **(A)** AGS-EBV cells were treated with miRNA inhibitors against miR-409-3p, miR-381-3p, miR-370-3p, or a pool of all three inhibitors (All 3) or a negative control (CTL). Cells were then reactivated to the lytic cycle with NaB treatment for 20 hrs, lysed in 9 M urea and analysed by Western blotting using antibodies against BZLF1, VCAP18 and actin (loading control). BZLF1 (grey), and VCAP18 (black) bands were quantified from 5 experiments relative to the control. Average values with standard deviation are shown on the right. **(B)** SNU719 cells were treated with miRNA inhibitors as in (A) then reactivated with NaB/TPA for 20 hrs. Lysates were analyzed by Western blotting using antibodies against BZLF1 (medium grey), BMRF1 (light grey), VCAP18 (black) and actin. Viral protein bands were quantified from four experiments relative to actin and plotted as in **A. (C)** HONE1-Akata cells were treated with individual miRNA mimics or indicated pools, lysed in 9 M urea and analysed by Western blotting using antibodies against ZEB1, BZLF1, and actin. ZEB1 (dark grey) and BZLF1 (light grey) bands were quantified from three experiments relative to actin and plotted as in **A. (D)** HONE1 cells were transfected with miRNA mimics of miR-409-3p, miR-381-3p, or negative-control miRNA mimic (CTL), followed by transfection with luciferase reporter containing WT BZLF1 promoter (Zp) or Zp with ZEB1 binding sites mutated (Mut), and a *Renilla* luciferase internal control plasmid. Firefly and *Renilla* luciferase were quantified, and firefly values were normalized to *Renilla* luciferase activity for each sample. For each luciferase assay, average values from three independent experiments (with standard deviation) are shown relative to the value for the negative control. * = 0.01 < P ≤ 0.05.

To determine the significance of the increase in miR-409-3p, miR-381-3p, and miR-370-3p miRNAs on EBV lytic infection, AGS-EBV cells were transfected with specific inhibitors targeting each miRNA, a pool of all three inhibitors, or a non-targeting control inhibitor. Four hours post-transfection, cells were reactivated with NaB treatment (for 20 hrs), then expression of the EBV BZLF1 (immediate early) and VCAP18 (late) proteins were examined by Western blotting. Inhibition of miR-409-3p and all three miRNAs resulted in a significant decrease in BZLF1, and VCAP18 expression (Fig 5A). This suggests that miR-409-3p promotes EBV lytic infection. We further investigated this effect of miR-409-3p in SNU719, a patient derived, naturally EBV-positive gastric carcinoma cell line [47]. Similar to the results in AGS-EBV, inhibiting miR-409-3p in SNU719 resulted in decreased levels of BZLF1, BMRF1 (early) and VCAP18 after EBV reactivation with NaB/TPA (Fig 5B).

miR-409-3p is known to target ZEB1 [48–50], a major negative regulator of EBV lytic reactivation and infection [31,32,51], and TargetScan predicts that miR-381-3p may also target ZEB1 [52]). Therefore, we wanted to determine if these miRNAs promoted EBV lytic infection by downregulating ZEB1. AGS-EBV cells are known to have very low levels of ZEB1, making them more prone to reactivation [33], and hence we could not reliably detect ZEB1 in these cells with available antibodies. Similarly, we could not detect ZEB1 in SNU719 cells. However, ZEB1 is readily detectable in HONE1-Akata cells, and therefore we used them to examine the effect of the EBV-induced miRNAs on ZEB1. HONE1-Akata were transfected with miRNA mimics of miR-409-3p, miR-381-3p, and miR-370-3p, individually or in combination, then ZEB1 proteins were examined by Western blotting (Fig 5C). miR-409-3p consistently decreased ZEB1 levels, while results with miR-381-3p, and miR-370-3p were inconsistent. In keeping with previous findings [31,51], the decrease in ZEB1 by the miR-409-3p mimic was accompanied by an increase in BZLF1. Combining miR-409-3p with miR-381-3p or with miR-381-3p and miR-370-3p had similar effects on the down regulation of ZEB1, suggesting that miR-409-3p is the main regulator of ZEB1.

To further examine the effects of miR-409-3p and miR-381-3p on BZLF1 expression through effects on ZEB1, we used these miRNA mimics in assays with a luciferase reporter controlled by the BZLF1 promoter, Zp, or a mutated version of Zp with mutated ZEB1 binding sites [33]. To this end, EBV-negative HONE1 cells were transfected with mimics of miR-409-3p, miR-381-3p, or negative control mimic, then with a Zp-luciferase reporter plasmid and a *Renilla* luciferase internal control. Both miR-409-3p and miR-381-3p significantly increased Zp activity compared to the control mimic (Fig 5D, left panel). This effect of the miR-409-3p mimic was abrogated when the same assay was performed with Zp with mutated ZEB1 sites (Fig 5D, right panel), suggesting that the miR-409-3p stimulated Zp through ZEB1. In contrast, the miR-381-3p mimic retained some ability to stimulate Zp with mutated ZEB1 binding sites, suggesting that this miRNA has additional targets impacting Zp.

### The DLK1-DIO3 locus transcripts increase upon EBV lytic infection

**Fig 6:**
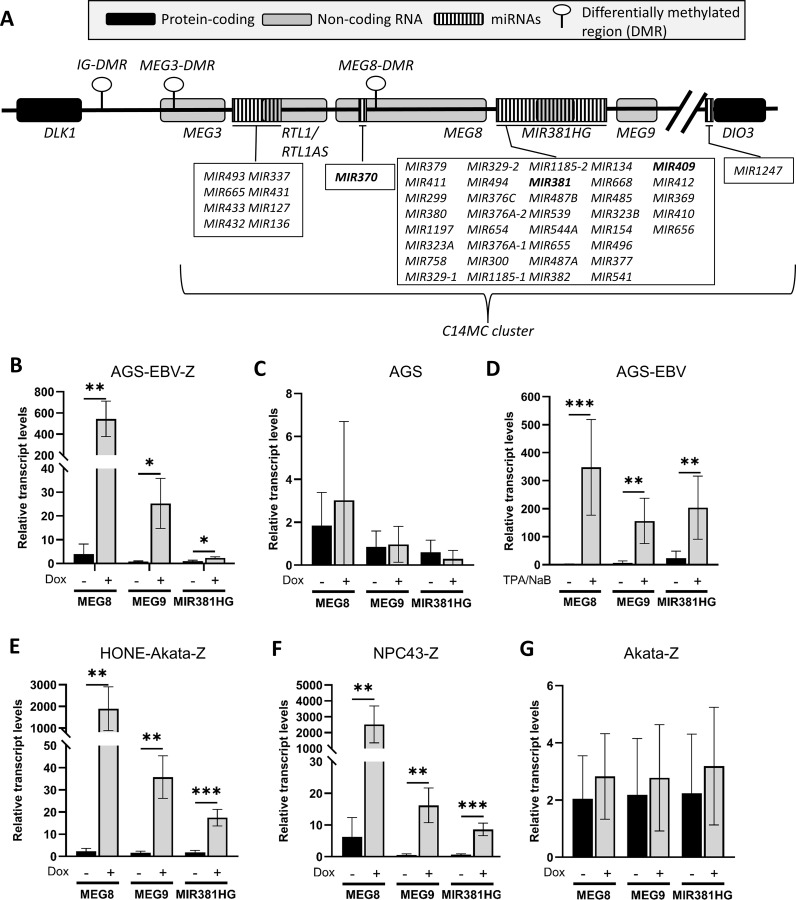
MEG8, MEG9, and MIR381HG transcripts increase upon EBV reactivation. **(A)** Schematic of the DLK1-DIO3 locus containing the chromosome 14 miRNA cluster (C14MC), showing the positions of the genes, the encoded miRNAs and the IG-DMR regulatory element. miRNAs identified in our miRNA-seq data are bolded. **(B)** Total RNA was isolated from AGS-EBV-Z cells, before or after reactivation with dox, and transcripts for MEG8, MEG9, and MIR381HG were quantified by RT-qPCR, relative to GAPDH transcripts. Average values with standard deviations are shown **(C)** Total RNA was isolated from AGS cells, before or after dox treatment, then transcripts were quantified as in **B. (D)** Total RNA was isolated from AGS-EBV cells before and after TPA/NaB treatment, then transcripts were quantified as in B **(E)** Total RNA was isolated from HONE-Akata-Z cells, before or after dox treatment, then transcripts were quantified as in **B. (F)** Total RNA was isolated from NPC43-Z cells, before or after dox treatment, then transcripts were quantified as in **B. (G)** Total RNA was isolated from and Akata-Z cells, before or after dox treatment, then transcripts were quantified as in **B.** All experiments were performed at least three times. * = 0.01 < P ≤ 0.05, ** = 0.001 < P ≤ 0.01, *** = 0.0001 < P ≤ 0.001.

One reason that miRNA levels might increase is if transcription of their RNA precursors is upregulated. Interestingly, the miRNAs that are most increased in lytic infection (miR-370-3p, miR-409-3p, and miR-381-3p) are all derived from the chromosome 14 miRNA cluster (C14MC) within the DLK1-DIO3 locus (14q32.2-32.31; Fig 6A). Multiple lncRNAs that give rise to miRNAs are expressed from the DLK1-DIO3 locus*,* such as *MEG8* (maternally expressed gene 8) that includes miR-370-3p, *MIR381HG* that includes miR-381-3p and *MEG9* that includes miR-409-3p [53]. Therefore, we investigated whether EBV lytic reactivation upregulated *MEG8, MEG9,* and *MIR381HG* transcripts. To this end, AGS-EBV-Z were treated with dox or left untreated, then 24 hrs later, *MEG8, MEG9,* and *MIR381HG* transcripts were quantified by RT-qPCR (normalized to *gapdh* transcripts). All of these transcripts were found to significantly increase upon lytic reactivation relative to latently infected cells (Fig 6B). This increase was not due to dox treatment, as EBV-negative AGS cells treated with dox showed no significant change in transcripts (Fig 6C). In addition, AGS-EBV cells reactivated to the lytic cycle with TPA/NaB also had greatly increased levels of *MEG8, MEG9,* and *MIR381HG* (Fig 6D), showing that the increase in these transcripts occurs regardless of the method of reactivation.

We then determined whether the increase in *MEG8, MEG9* and *MIR381HG* upon EBV reactivation occurred in other EBV-positive cell lines. Similar to AGS-EBV-Z cells, reactivation of HONE1-Akata-Z and NPC43-Z nasopharyngeal carcinoma cell lines led to greatly increased levels of *MEG8, MEG9,* and *MIR381HG* (Fig 6E and 6F, respectively). We also investigated whether the increase in lncRNAs occurred upon lytic infection in B cells, using the EBV-positive Burkitt’s lymphoma cell line, Akata, containing a dox-inducible BZLF1 (Akata-Z). Unlike the EBV-positive epithelial cell lines, *MEG8, MEG9,* and *MIR381HG* transcripts did not increase significantly after the reactivation of Akata-Z cells (Fig 6G). Together, the results suggest that induction of lncRNAs at the C14MC/DLK1-DIO3 locus is responsible for the increase in miR-370-3p, miR-409-3p, and miR-381-3p in lytic infection and that this effect is specific to epithelial cells.

### BZLF1 is responsible for induction of lncRNAs from the DLK1-DIO3 locus during lytic infection

**Fig 7:**
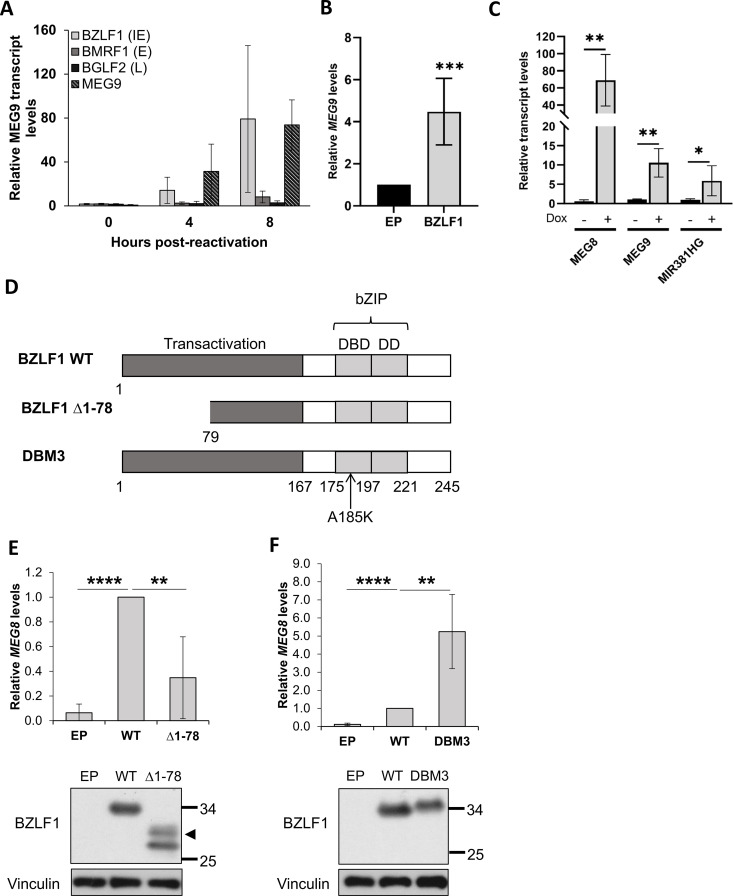
EBV BZLF1 induces DLK1-DIO3 transcripts. **(A)** Total RNA was isolated from AGS-EBV cells treated with TPA/NaB for 0, 4 or 8 hrs, then transcripts for *MEG9* and EBV BZLF1, BMRF1, and BGLF2 were quantified by RT-qPCR. mRNA expression was normalized to GAPDH transcripts and shown relative to 0 hr samples. **(B)** AGS cells were transfected with a plasmid expressing BZLF1 or empty plasmid (EP) control. Twenty-four hours later, RNA was isolated and *MEG9* transcripts were quantified by RT-qPCR, normalized to GAPDH transcripts and shown relative to the EP control. **(C)** Total RNA was isolated from AGS-Z cells before and after dox treatment, and *MEG8*, *MEG9*, and *MIR381HG* transcripts were quantified by RT-qPCR, normalized to GAPDH transcripts and shown relative to untreated cells. **(D)** Schematic of BZLF1, and BZLF1 transactivation and DNA binding mutants. **(E and F)** HONE1 cells were transfected with plasmids expressing HA-tagged BZLF1 or ∆1-78 BZLF1 mutant (E) or with plasmids expressing BZLF1 or DBM3 BZLF1 mutant (F) or with the corresponding empty plasmid (EP). *MEG8* transcripts were quantified by RT-qPCR, normalized to actin transcripts, and shown relative to values for WT BZLF1 (set to 1). Western blots showing expression of the BZLF1 proteins are shown under each graph. The arrowhead indicates the position of full length Δ1-78. All experiments were performed at least three times. * = 0.01 < P ≤ 0.05, ** = 0.001 < P ≤ 0.01, *** = 0.0001 < P ≤ 0.001, **** = P ≤ 0.0001.

To better understand the cause of the increase in transcripts from the DLK1-DIO3 locus upon EBV lytic reactivation, we first characterized the timing of *MEG9* induction during lytic infection to determine what temporal class of EBV protein might be responsible. To this end, AGS-EBV cells were reactivated to the lytic cycle with TPA/NaB and *MEG9* mRNA was quantified by RT-qPCR before and at various time points after reactivation, then compared to transcripts for EBV immediate early (*bzlf1*), early (*bmrf1*), and late (*bglf2*) transcripts. *MEG9* levels were increased at 4 hrs post-reactivation, a time point at which *bzlf1*, but not *bmrf1* or *bglf2*, was also increased and further increased along with *bzlf1* expression at 8 hrs post-reactivation (Fig 7A). The expression pattern suggests that an immediate early event or protein, such as BZLF1, triggers the increase in *MEG9* transcripts.

Since BZLF1 is an immediate-early protein that can act as a transcriptional regulator (reviewed in [54]), we tested whether it could induce *MEG9* transcripts. This was done in two ways, first by transiently expressing BZLF1 in AGS cells (Fig 7B), and secondly by using AGS cells containing a dox-inducible BZLF1 (AGS-Z) and treating with dox for 48 hrs (Fig 7C). In both cases, *MEG9* transcripts were significantly increased upon expression of BZLF1. The effects of BZLF1 expression on *MEG8* and *MIR381HG* transcripts in the AGS-Z cells were also examined and both were found to be significantly increased (Fig 7C). The results suggest that BZLF1 is responsible for the induction of the DLK1-DIO3 locus transcripts that give rise to miR-409-3p, miR-381-3p, and miR-370-3p miRNAs.

BZLF1 transactivates gene expression using its N-terminal transactivation domain and can be recruited to specific recognition sites in DNA (called ZREs) through its C-terminal DNA binding domain [55–58]. To determine whether induction of DLK1-DIO3 locus transcripts by BZLF1 involves its transactivation or DNA binding activities, we compared the effects of WT BZLF1 to mutants lacking the first 78 amino acids of the transactivation domain (∆1–78; Fig 7D and 7E) or disrupted in DNA binding by a A185K point mutation (DBM3; Fig 7D and 7F; [56]) on *MEG8* induction (the most highly induced transcript). Transient expression of these BZLF1 proteins in HONE1 cells showed that induction of *MEG8* was dependent on the transactivation domain but not on the DNA binding activity of BZLF1. In fact, the BZLF1 DNA binding mutant consistently showed increased induction of *MEG8* (Fig 7F), which might indicate that the inability to be recruited to ZREs resulted in it being more available to transactivate *MEG8.*

### BZLF1 interacts with a key control region of the DLK1-DIO3 locus

**Fig 8:**
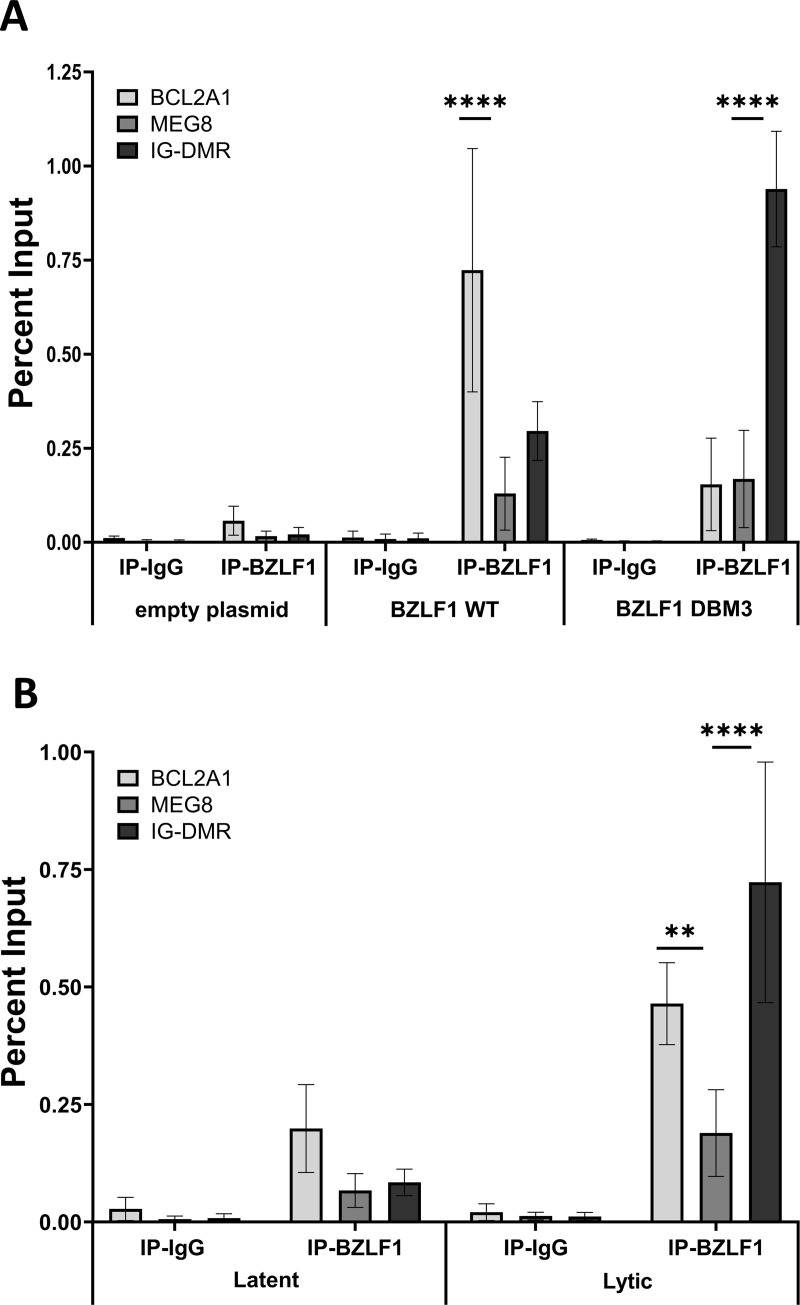
BZLF1 associates with the IG-DMR in the DLK1-DIO3 locus. **(A)** AGS cells were transfected with a plasmid expressing WT BZLF1, or the BZLF1 DBM3 mutant or with empty plasmid, followed by formaldehyde cross-linking 24 hrs later. ChIP was performed on sheared chromatin using BZLF1 or negative control IgG_1_ antibodies, and associated DNA from the IG-DMR (black bars), a negative control MEG8 region (dark grey bars), or a positive control BCL2A1 region (light grey) were quantified by qPCR and shown as percent of input DNA. **(B)** AGS-EBV-Z cells were reactivated for 24 hrs with dox (lytic) or left untreated (latent), then ChIP-qPCR was performed and data displayed as in **(A)**. For both sets of experiments, average values from three independent experiments are shown along with their standard deviation. ** = 0.001 < P ≤ 0.01, **** = P ≤ 0.0001.

The finding that BZLF1 can induce expression of *MEG8*, *MEG9* and *MIR381HG,* raises the possibility that it might be interacting with control elements in the DLK1-DIO3 locus to activate the expression of these lncRNAs. The intergenic differentially methylated region (IG-DMR; shown in Fig 6A) of the DLK1-DIO3 locus has been shown to play a major role in regulating gene expression from this locus, including *MEG8*, *MEG9* and *MIR381HG.* Therefore, we performed chromatin immunoprecipitation (ChIP) experiments to investigate the association of BZLF1 with this DLK1-DIO3 control region. Initially, we expressed BZLF1 or the DBM3 BZLF1 DNA binding mutant in AGS cells and performed ChIP experiments using antibody specific to BZLF1 or IgG isotype negative. Recovery of the IG-DMR region was determined and compared to that of a known BZLF1 interaction site in the *BCL2A1* gene promoter as a positive control [59], and to region in the middle of *MEG8* as a negative control. As expected, WT BZLF1 ChIP’d to the BCL2A1 promoter but the BZLF1 DBM3 did not, and neither BZLF1 protein associated with the MEG8 fragment (Fig 8A). WT BZLF1 also showed some enrichment at the IG-DMR region relative to the MEG8 region, and this association was greatly increased with the DBM3 mutant (Fig 8A, black bars). This indicates that BZLF1 associates with the IG-DMR but that this does not require direct DNA binding. It also suggests that disrupting the DNA binding ability of BZLF1 increases the availability of BZLF1 to associate with the IG-DMR.

We then examined whether BZLF1 associates with IG-DMR during EBV infection. To this end, AGS-EBV-Z cells were either left in latency or reactivated using dox, followed by ChIP with antibodies against BZLF1 or IgG isotype control, and recovery of *BCL2A1, MEG8,* and IG-DMR DNA regions were analyzed (Fig 8B). In lytic infection, BZLF1 was found to associate with BCL2A1, as expected, but showed an even greater association with IG-DMR, suggesting that BZLF1 might promote expression of transcripts from this locus by acting at this control region.

## Discussion

Several studies have shown that the expression of both cellular and EBV miRNAs varies in different types of latency. These include miRNA-seq studies comparing EBV-positive and EBV-negative AGS [40], BL [38,39,41,42], NPC [43], HL [43], and DLBCLs [37]. However, much less is known about differences in miRNA expression between latent and lytic EBV infections. In this study, we have provided a comprehensive analysis of changes in miRNA expression and Ago2-association in latent and lytic infections in AGS-EBV cells. Prominent findings include that lytic reactivation results in induction of the EBV BHRF1 miRNAs, miR-BHRF1–2-3p and miR-BHRF1–3, which were not previously known to be expressed in EBV-positive gastric carcinoma cells, and induction of specific miRNAs from the C14MC locus.

Analysis of input and Ago2-association in AGS cells showed that 31/44 EBV miRNAs are expressed and utilized in latent and lytic infections. These miRNAs are largely derived from the BART clusters 1 and 2. This is in contrast to a study in BL cells, in which the levels of BART miRNAs were unchanged after lytic reactivation [37]. miR-BARTs have been shown to have important roles in promoting lytic infection. miR-BART6-5p, miR-BART6-3p, miR-BART18-5p and miR-BART-20-5p have all been shown to regulate BZLF1 and BRLF1 [17,60,61]. Conversely, miR-BART9 can promote lytic infection by mimicking the role of cellular miR-141 in downregulating B cell transcription factors and negative regulators of immediate early genes [62]. In our study, the fold increase in different BART miRNAs upon EBV reactivation varied considerably, with miR-BART7-5p showing the highest fold increase (~40-fold). While multiple studies found that miR-BART7-3p is one of the highest expressed EBV miRNAs in multiple human tumours and latently infected B cell lines [63–70], miR-BART-7-5p was one of the least prevalent BART miRNAs in these studies [64–66,68]. Thus, miR-BART-7-5p may have roles specific to EBV lytic infection.

In addition, we found that miR-BHRF1–2-3p and miR-BHRF1–3, which were lowly expressed in latent infection, were highly induced in lytic infection. This was unexpected as BHRF1 miRNAs are typically associated with latency III B cell infections [71]. Interestingly, Fachko *et al.* [18] showed that EBV miR-BHRF1–3 targets the 3′ UTR of *bzlf1* mRNA, decreasing the level of BZLF1 and therefore downregulating lytic infection. Therefore, it is likely that miR-BHRF1–3 plays a similar role in attenuating lytic infection in AGS cells, although there may be additional targets.

We also identified 107 cellular miRNAs that are differentially expressed in lytic infection in AGS cells compared to latent infection. miR-107, a member of the miR-16 family, was the most decreased upon EBV reactivation (~7-fold decrease). Previous study by Godshalk *et al.* [72] also identified miR-107 to be significantly decreased when 293 cells containing an EBV bacmid were reactivated to lytic infection. Interestingly, the *MEG8* transcript of the DLK1-DIO3 locus, which we showed was induced upon EBV reactivation, acts as a sponge for miR-107, so might be at least partially responsible for the decrease in miR-107 [73,74]. Additional miRNAs that we found to be downregulated in lytic infection include miR-31-5p, miR-29a-3p, miR-181-3p, miR-194-5p were recently reported to be downregulated in KSHV lytic infection [75], suggesting that the downregulation of these miRNAs might be important for gamma-herpesviruses to maintain latency.

This study identified miR-409-3p, miR-381-3p, and miR-370-3p as highly increased in abundance and Ago2-association upon lytic reactivation in multiple carcinoma cell lines. Interestingly, these miRNAs are derived from lncRNAs expressed from the same chromosomal locus, DLK1-DIO3 found on chromosome 14q32.2-32.31 (GRCh37.p13) [76]. This ~1 Mbp imprinted locus encodes coding and non-coding RNA species including lncRNAs, C/D small nucleolar RNAs (SNORDs), and miRNAs, and has been shown to be important for embryogenesis and fetal development [77–80]. The locus has allele-specific expression due to genomic imprinting, including differences in DNA methylation on each allele [81–86]. Most of the miRNAs within the DLK1-DIO3 locus reside within the C14MC cluster as two genomic regions: the miR-127/miR-136 cluster and the miR-370/miR-410 (or miR-379/miR656) cluster. miR-409-3p and miR-381-3p are found in the latter cluster, while miR-370-3p is found outside these clusters and overlaps with *MEG8.* C14MC encodes more than 50 miRNAs, making it one of the largest miRNA clusters found in the human genome. C14MC expression typically occurs in the placenta and embryos, with detection in adults limited to the brain [9,77,80,87]. Increased expression of C14MC miRNAs has been associated with relapsing-remitting multiple sclerosis (RRMS) in men [88], cervical carcinoma [89], lung adenocarcinomas [90] and other non-malignant respiratory diseases [91]. Predicted targets of miRNAs from the C14MC cluster are enriched in KEGG pathway terms for EBV and other viral infections [89], suggesting that they may play roles in viral infections. Consistent with this hypothesis, we have shown that miR-409-3p and miR-381-3p have some properties consistent with roles in promoting lytic EBV infection.

miR-409-3p, miR-381-3p, and miR-370-3p have multiple predicted targets. Interestingly, both miR-409-3p and miR-381-3p are predicted to target ZEB1 [52], and miR-409-3p has been confirmed to target ZEB1 in multiple cancer cells [48–50,92]. ZEB1 is known to repress BZLF1 expression and hence lytic infection, through a direct interaction with Zp [31–34]. Here we confirmed downregulation of ZEB1 by miR-409-3p in HONE1-Akata cells. We also found that both miR-409-3p and miR-381-3p increased activity of Zp, although only the activity of miR-409-3p was completely dependent on the ZEB1 binding sites, suggesting that the induction of the DLK1-DIO3-derived miRNAs promotes BZLF1 expression, in part through effects on ZEB1. miR-200b and miR-429 have been previously shown to promote EBV reactivation by downregulating ZEB1 and ZEB2 [33,34]. Although we did not see changes in miR-200b and miR-429 levels upon reactivation (S1 Table), these miRNAs were highly abundant and Ago2-associated in lytic infection in AGS cells (S1 and S2 Tables), suggesting that they might play roles in lytic infection.

In addition to the increase in miR-409-3p, miR-381-3p, and miR-370-3p upon EBV reactivation, we have found that the transcripts from which they are derived within the DLK1-DIO3 locus are also induced, suggesting that increased transcription might account for the increase in these miRNA levels. There may also be regulation at the level of processing and maturation of pre-miRNAs and miRNAs, which may explain why only miR-409-3p, miR-381-3p, and miR-370-3p were increased while other miRNAs from the same initial transcripts were undetected. While an increase in *MEG8, MEG9* and *MIR381HG* miRNA precursors was consistently seen in epithelial-derived cell lines, we did not see induction of these lncRNAs upon reactivation of Burkitt’s lymphoma cell lines (Fig 6G), suggesting that the activation of transcripts in the DLK1-DIO3 locus is specific to EBV infection of epithelial cells. We hypothesize that, in B cell lines, the common translocation events occurring at the IGH locus on chromosome 14 (reviewed in [93]) may be impairing the expression of transcripts from the DLK1-DIO3 locus. Previous work has shown that translocation events at this locus in B cell malignancies cause hypermethylation of the DLK1-DIO3 locus [94], and it may be that this hypermethylation prevents expression of the lncRNAs. This is also consistent with a previous study profiling miRNAs in reactivated Burkitt’s lymphoma cells (MutuI; which also has a translocation on chromosome 14 [95]), which did not detect miRNAs from the DLK1-DIO3 locus [62].

**Fig 9:**
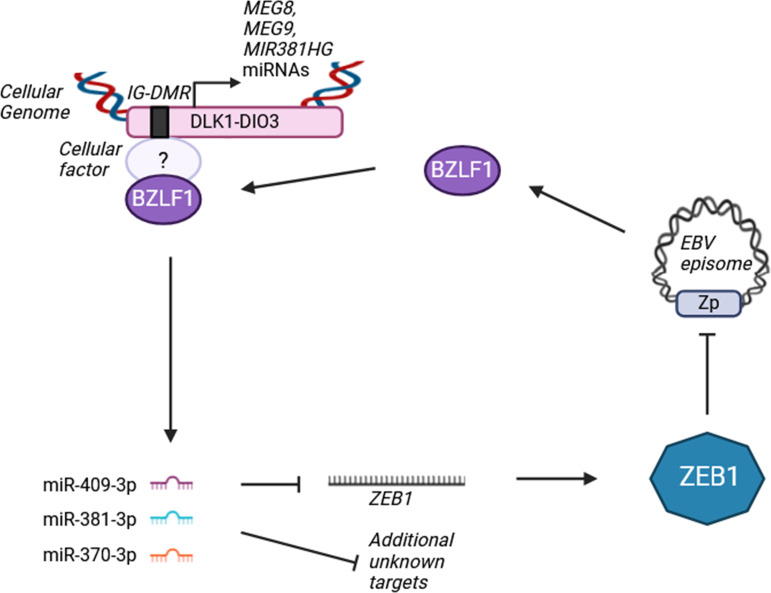
Model of miRNA induction by BZLF1. In lytic infection, BZLF1 associates with the IG-DMR in the DLK1-DIO3 locus to activate the transcription of lncRNAs, including *MEG8*, *MEG9* and *MIR381HG*, which give rise to miR-409-3p, miR-381-3p and miR-370-3p. These miRNAs increase BZLF1 expression and lytic infection, in part by targeting ZEB1.

We have shown that EBV BZLF1 is sufficient for induction of the DLK1-DIO3 transcripts. BZLF1 is known to function as a transcriptional activator for some EBV genes and may have similar roles in activating the expression of some cellular genes [96]. We found that induction of *MEG8* transcripts by BZLF1 requires its transactivation domain, consistent with a role in activating transcription. ChIP experiments also showed that BZLF1 associated with the IG-DMR regulatory element of the DLK1-DIO3 locus, suggesting that BZLF1 directly activates expression of transcripts from this locus. IG-DMR is an imprinted control region (ICR) which regulates paternal- or maternal-specific gene expression through DNA methylation status, resulting in expression of lncRNAs and miRNAs from the unmethylated maternal strand [97]. While BZLF1 was found to associate with the IG-DMR and induce *MEG8*, neither property required its DNA binding activity, suggesting that BZLF1 is recruited to the IG-DMR control element through an interaction with another protein (Fig 9). For example, TRIM28 has been shown to interact with IG-DMR where it maintains DNA methylation of the paternal strand [97–100]. Since BZLF1 interacts with TRIM24/28/33 complexes [44], this could be one way BZLF1 is recruited to the DLK1-DIO3 locus.

Our results support a model in which, in EBV lytic infection in epithelial cells, BZLF1 interacts with and transactivates genes within the DLK1-DIO3 locus, resulting in an increase in miR-409-3p, miR-381-3p, and miR-370-3p (Fig 9). These miRNAs then promote lytic infection, in part through downregulation of ZEB1. Thus, our study has revealed an additional mechanism by which BZLF1 promotes the lytic cycle.

## Materials and methods

### Cell lines

AGS-EBV-Z, AGS-BZLF1, SNU719, Akata-Z, HONE-1 and NPC43-Z cells were all previously described [44,47,101,102]. HONE-Akata-Z cells were derived from the EBV-positive HONE1-Akata [102] cells, which were originally derived from nasopharyngeal carcinoma cells, but have been demonstrated to contain sequences from HeLa cells as well as HPV18 [103,104]. HONE-Akata-Z cells were generated by transducing HONE1-Akata cells, grown in RPMI+10% FBS in a 6-well plate, with lentivirus made from pTRIPZ plasmid containing a BZLF1 expression cassette (pTRIPZ-BZLF1) [101]. Twenty-four hours post-transduction, puromycin was added to 2 μg/ml to select for cells containing the cassette.

All cell lines of AGS origin, SNU719, NPC43-Z and Akata cell lines were cultured in RPMI (Multicell 350–000-CL) medium while HONE1-Akata cell lines were cultured in AMEM medium (Multicell; 310–010-CL). All media was supplemented with 10% fetal bovine serum (FBS; Multicell 098150) and 1% penicillin/streptomycin (Gibco 15140–122). NPC43-Z cells were also supplemented with 4 μM ROCK inhibitor (Enzo Y-27632). AGS-EBV-Z cells were also supplemented with 400 μg/ml G418 (Geneticin; Gibco 10131–027) and all Tet-inducible BZLF1 cell lines were supplemented with 2 μg/ml puromycin.

### Plasmids

Reporter plasmids with luciferase gene under control of the BZLF1 promoter, Zp (pZp-luc), and ZP lacking ZEB1 binding sites (pZp-triple mutant-luc) were a gift from Dr. Janet Mertz and were previously described [33]. pBS(SVp/e) plasmids expressing WT BZLF1 or the BZLF1 DBM3 DNA binding mutant or HA-tagged BZLF1 Δ1–78 mutant were a gift from Dr. Zhen Lin and are described in Zhao et al. [56]. To generate a plasmid expressing HA-tagged WT BZLF1, pBS(SVp/e) containing WT BZLF1 was digested with MlsI and an oligo duplex containing the HA sequence (ATG TAT CCT TAT GAT GTG CCT GAC TAT GCC and GGC ATA GTC AGG CAC ATC ATA AGG ATA CAT) was phosphorylated then ligated with the MlsI-digested plasmid.

### miRNA library preparation and sequencing

AGS-EBV-Z cells were seeded in two 15-cm dishes at ~60% confluency. Twenty-four hours later, BZLF1 gene expression was induced with 2 μg/ml doxycycline (dox) in one of the 15-cm plates (lytic infection). The remaining plate was left untreated (latent infection). Cells were lysed 48 hrs post-dox treatment in 600 μl lysis buffer (25 mM Tris-HCl pH 7.4, 150 mM KCl, 2 mM EDTA, 0.5% NP-40 substitute, complete protease inhibitors) for 30 min at 4°C and lysates were clarified by centrifugation. Samples of the clarified lysate were removed for protein (10 μl) and RNA (15 μl) analyses. The remaining clarified lysate was pre-cleared by 1 hr incubation with 50 μl protein A/G magnetic beads at 4°C as described previously [102]. Lysates were then divided in two and incubated with either Ago2- or IgG2α-conjugated magnetic beads overnight at 4°C with rotation. Conjugated beads consisted of 100 μg protein A/G magnetic beads (GB-Magnetic; 2220213) coupled to 1 μg rat anti-Ago2 (clone 11A9, Millipore; MABE253) or rat IgG2a (Millipore; MABF1077Z). Beads were washed 3 times with Ago2-IP washing buffer (50 mM Tris-HCl pH 7.4, 300 mM KCl, 1 mM MgCl2, 0.5% NP-40 substitute) and then resuspended in 500 μl PBS. 25 μl of each sample in PBS was removed for Western blotting to quantify Ago2 recovery. The remaining beads and 5 μl of cleared lysate were suspended in 1 ml NucleoZol for RNA isolation. RNA concentration was determined by NanoDrop (Thermo Scientific).

RNA was isolated from the input and Ago2-IP samples using Nucleozol, following the manufacturer’s protocols. cDNA libraries were generated using the NEBNext Small RNA Library Prep Set for Illumina (Multiplex Compatible; E7330). Library quality control and sequencing was performed by The Centre for Applied Genomics (TCAG, Toronto, Ontario, Canada). Sequencing was performed on an Illumina NovaSeq – SP flow cell with an expected coverage of 10 million reads per sample. Sequencing was aligned to the human (GRCh38.p13; GCF_000001405.39) and EBV Akata (KC207813.1) genomes using miRDeep2 ([105]; https://github.com/rajewsky-lab/mirdeep2) and differential expression was analyzed using EdgeR [106,107]. The raw data has been deposited in NCBI sequence read archive (SRA) as BioProject PRJNA1282670.

### RNA isolation and quantification by RT-PCR

All cell lines, except Akata-Z, were seeded in 10 cm dishes at ~60% confluency. Twenty-four hours later, cells were either treated with 2 μg/ml dox to induce BZLF1 expression for 24 hrs, 48 hrs or left untreated. For Akata-Z cells, 30 ml of confluent cells were seeded in 75 mm^2^ flasks and immediately treated with 2 μg/ml dox for 24 hrs. Cells were lysed and processed for mRNA or miRNA analysis. miRNA was isolated using the *mir*Vana miRNA Isolation Kit, with phenol (ThermoFisher; Cat: AM1560) and resuspended in 50 μl of RNase-free water.

miRNA cDNA and real-time PCR were performed on miRNA-enriched RNA following the miRCURY LNA miRNA PCR kit (Qiagen) protocol. Briefly, 100 ng of each miRNA-enriched RNA sample was combined with 5 ng UniSp6 (spike-in control), and then reverse transcription was performed. The resulting cDNA was diluted 10-fold and 3 μl was used for PCR with primers targeting specific miRNAs as follows: hsa-miR-409-3p (YP00204358), hsa-miR-381-3p (YP00205887), hsa-miR-370-3p (YP00204011), hsa-miR-151a-3p (YP00204576), and UniSp6 (supplied with PCR kit). All Cq values were normalized to miR-151a-3p, and fold changes were calculated relative to latent infection.

For EBV-derived miRNAs, cDNA synthesis and real-time PCR were performed on miRNA enriched RNA following the miR-X miRNA First-Strand Synthesis and TB Green qRT-PCR (Takara) protocol. Up to 10 µg of small RNA was treated with DNase I (RNase-free; New England Biolabs Cat No. M0303) following manufacturer’s protocol and used for reverse transcription. Primers for ebv-miRNAs were designed (see Table 3) to match each miRNA sequence on miRbase [108–112]. All Cq values were normalized to miR-151a-3p, and fold changes were calculated relative to latent infection. The relative miRNA expression was derived from 2^-ΔΔCT^ by use of the comparative threshold cycle (CT) method.

**Table 3:** Primers used in this study.

Primers for transcripts	Direction	Sequence (5′ → 3′)	References
MEG9	Forward	CACCCAGTGCTGCTTTTGAC	This paper
	Reverse	CCGGGGACCAGAAGGTAAAC	
MEG8	Forward	CAGTGTTGCCTGGGTCTGA	[113]
	Reverse	ATCCCCTTGAAAGAGCAGGA	
MIR381HG	Forward	ATCCTCAGCCCAAGGAAAGC	This paper
	Reverse	TGGCCATGAAGCCATGCTAA	
Actin	Forward	GGACTTCGAGCAAGAGATGG	[114]
	Reverse	AGCACTGTGTTGGCGTACAG	
GAPDH	Forward	GAGTCAACGGATTTGGTCGT	[36]
	Reverse	AATGAAGGGGTCATTGATGG	
BZLF1	Forward	CATGTTTCAACCGCTCCGACTGG	[115]
	Reverse	GCGCAGCCTGTCATTTTCAGATG	
BMRF1	Forward	CTAGCCGTCCTGTCCAAGTGC	[115]
	Reverse	AGCCAAACAGCTCCTTGCCCA	
BGLF2	Forward	ACTGCCCACGTCTTTACCAC	This paper
	Reverse	GGCACCATAGCATGTCACAC	
Primers for miRNAs			
hsa-miR-151a-3p	Forward	CTAGACTGAAGCTCCTTGAAG	This paper
ebv-miR-BART6-5p	Forward	TAAGGTTGGTCCAATCCATAGG	This paper
ebv-miR-BART11-3p	Forward	ACGCACACCAGGCTGACTGCC	This paper
ebv-miR-BART4-3p	Forward	CACATCACGTAGGCACCAGGTGT	This paper
ebv-miR-BART4-5p	Forward	GACCTGATGCTGGTGTGCT	This paper
ebv-miR-BART1-3p	Forward	TAGCACCGCTATCCACTATGTC	This paper
ebv-miR-BART2-5p	Forward	TATTTTCTGCATTCGCCCTTGC	This paper
ebv-miR-BART9-3p	Forward	TAACACTTCATGGGTCCCGTAGT	This paper
ebv-miR-BART6-3p	Forward	CGGGGATCGGACTAGCCTTAGG	This paper
ebv-miR-BART13-3p	Forward	TGTAACTTGCCAGGGACGGCTGA	This paper
ebv-miR-BART18-5p	Forward	TCAAGTTCGCACTTCCTATACA	This paper
ebv-miR-BART11-5p	Forward	TCAGACAGTTTGGTGCGCTAGTTG	This paper
ebv-miR-BART17-3p	Forward	TGTATGCCTGGTGTCCCCTTAGT	This paper
ebv-miR-BART19-5p	Forward	ACATTCCCCGCAAACATGACATG	This paper
ebv-miR-BART17-5p	Forward	TAAGAGGACGCAATACAAG	This paper
ebv-miR-BART8-5p	Forward	TACGGTTTCCTAGATTGTACAG	This paper
ebv-miR-BART12	Forward	TCCTGTGGTGTTTGGTGTGGTT	This paper
ebv-miR-BART1-5p	Forward	TCTTAGTGGAAGTGACGTGCTGTG	This paper
ebv-miR-BART14-3p	Forward	TAAATGCTGCAGTAGTAGGGAT	This paper
ebv-miR-BART14-5p	Forward	TACCCTACGCTGCCGATTTACA	This paper
ebv-miR-BART16	Forward	TTAGATAGAGTGGGTGTGTGCTCT	This paper
ebv-miR-BART7-5p	Forward	CCTGGACCTTGACTATGAAACA	This paper
ebv-miR-BART22	Forward	TTACAAAGTCATGGTCTAGTAGT	This paper
ebv-miR-BART13-5p	Forward	AACCGGCTCGTGGCTCGTACAG	This paper
ebv-miR-BART19-3p	Forward	TTTTGTTTGCTTGGGAATGCT	This paper
ebv-miR-BART8-3p	Forward	GTCACAATCTATGGGGTCGTAGA	This paper
ebv-miR-BART10-3p	Forward	TACATAACCATGGAGTTGGCTGT	This paper
ebv-miR-BART7-3p	Forward	CATCATAGTCCAGTGTCCAGGG	This paper
ebv-miR-BART18-3p	Forward	TATCGGAAGTTTGGGCTTCGTC	This paper
Universal miR-X reverse primer	Reverse	Provided by the manufacturer	
Primers for ChIP-qPCR			
BCL2A1	Forward	TCTTGAGCTGGCTCACCTTG	[59]
	Reverse	AAACACAGCCTACGCACGAA	
MEG8	Forward	GCTAACCCGTAACCAAGGCT	This paper
	Reverse	TACTTTGGGCACCACGAGAC	This paper
IG-DMR	Forward	AGCGATTTGCCAATTGCGAG	This paper
	Reverse	GGAAGCCTAAAGGGCGAGTC	This paper

Cell lines with dox-inducible BZLF1 were treated as above. AGS-EBV, cells were treated with 3 mM sodium butyrate (NaB) and 20 ng/ml 12-O-tetradecanoylphorbol-13-acetate (TPA) 24 hrs prior to lysis. Total RNA was isolated from cell lysates using NucleoZol (TaKaRa Bio) according to the manufacturer’s instructions. RNA was prepared as described previously [102] for use with the Luna Universal One-Step RT-qPCR kit (New England BioLabs), with a total reaction volume of 10 μl in a Bio-Rad CFX384 Real-Time System (Bio-Rad). Primers used for this study are described in Table 3. The relative RNA expression was derived from 2^-ΔΔCT^ by use of the comparative threshold cycle (CT) method. The abundance of mRNA in each sample was normalized to the amount of GAPDH mRNA.

### Effects of BZLF1 and mutants on *MEG8*

AGS and HONE1 cells were seeded in 6-well plates at ~2.5 x 10^5^ cells per well. Twenty-four hours later, cells at 70% confluency were transfected with 2 μg pBS(SVp/e) plasmid expressing HA-tagged WT BZLF1 or ∆1–78, or untagged WT BZLF1 or DNA-binding mutant (DBM3) or empty plasmid control [56] using lipofectamine 2000:DNA at a 2:1 ratio. Cells were harvested 24 hrs later, and RNA was isolated using NucleoZol following the manufacturer’s instructions.

### miRNA inhibitor and mimic experiments

AGS-EBV and SNU719 cells were seeded in 6-well plates. Twenty-four hours later, cells at 70% confluency were transfected with 90 pmol of *miR*Vana miRNA inhibitor Negative Control #1 (ThermoFisher, Cat no: 4464076), or inhibitors of hsa-miR-409-3p (Cat no: 4464084, Assay ID: MH12446), hsa-miR-381-3p (MH10242), hsa-miR-370-3p (MH12868), or 30 pmol of all three inhibitors using 2.5 μl RNAiMAX (ThermoFisher Scientific, Cat no: 13778075) per well. After 4 hrs, cells were treated with 3 mM NaB, with (SNU719) or without (AGS-EBV) 20 ng/ml TPA, then lysed in 9M urea, 10 mM Tris pH 6.8 20 hrs later.

For miRNA mimics, HONE1-Akata cells were seeded in 6-well plates. Twenty-four hours later, cells at 70% confluency were transfected with 90 pmol of *miR*Vana miRNA mimic Negative Control #1 (Cat no: 4464058), or mimics of hsa-miR-409-3p (Cat no: 446066; Assay ID: MC12446), hsa-miR-381-3p, (MC10242), hsa-miR-370-3p (MC12868), or 30 pmol of all three mimics using 2.5 μl RNAiMAX per well. For the combination of hsa-miR-409-3p and hsa-381-3p, 30 pmol of negative control inhibitor was used. After 24 hrs, cells were lysed in 9M urea, 10 mM Tris pH 6.8.

Thirty to one hundred micrograms of lysate was subjected to 12% SDS-PAGE and transferred to nitrocellulose. Membranes were blocked in 5% non-fat dry milk in PBS-T (phosphate-buffered saline (PBS) with 0.1% Tween) and then incubated with primary antibodies as indicated, including mouse anti-β-actin (Santa Cruz; sc-47778, 1:5,000–1:10,000), mouse anti-ZEBRA (BZLF1, Santa Cruz, sc-53904, 1:5000), mouse anti-BMRF1 (prepared from hybridoma kindly provided by Jaap Middeldorp), mouse anti-VCAP18 (prepared from hybridoma kindly provided by Jaap Middeldorp) and rabbit anti-ZEB1 (Cell Signaling; 3396, 1:500) overnight. Membranes were washed in PBS-T, followed by incubation with secondary antibodies goat anti-mouse horseradish peroxidase (HRP; Santa Cruz; sc-2005) or goat anti-rabbit HRP (Sigma-Aldrich; SAB3700853) at 1:5000 dilution for 1 hr. Membranes were washed in PBS-T and developed using Clarity ECL Western Blotting Substrates (BioRad; Cat no: 1705060) with Clonex BioFlex MSI Film, radiomat LB blue x-ray film (Mandel Scientific Company; XC6A2).

### Luciferase reporter assay

HONE1 cells were seeded in 6-well plates. Twenty-four hours later, cells at 70% confluency were transfected with 90 pmol of *miR*Vana miRNA mimic Negative Control #1, or mimics of hsa-miR-409-3p, or hsa-miR-381-3p, using 2.5 μl RNAiMAX per well. After 24 hrs, cells were transfected with 1.8 μg of pZp-luc and pZp-triple mutant-luc or pGL3 empty plasmid control, and 0.2 μg of pRL-TK [116] internal control for an additional 24 hrs. Luciferase assays were performed 24 hrs following the second transfection using a dual-luciferase reporter assay system (Promega) according to the manufacturer’s protocol. Firefly luciferase activity was normalized to internal *Renilla* luciferase activity for each sample. The average values from four independent experiments were normalized relative to the Negative Control #1 mimic value.

### Chromatin immunoprecipitation experiments

For ChIP experiments performed in EBV-negative cells, AGS cells were seeded into two 15-cm plates per condition. Twenty-four hours later, each plate of cells (at 80% confluency) was transfected with 11 μg pBS(SVp/e) plasmid expressing WT BZLF1, DBM3, or empty plasmid [56] using a 2:1 PolyJet:DNA ratio. Twenty-four hours post-transfection, cells were cross-linked with formaldehyde (1% final concentration in medium) for 15 min, then treated with glycine (0.125 M final concentration) for 5 min at room temperature with rocking. Cells were washed once and harvested by scraping in 5 ml ice-cold PBS, and then collected by centrifugation at 1710 g for 5 min. Cell pellets were lysed in 1 ml of ChIP Cell Lysis Buffer (85 mM KCl, 0.5% NP-40, 5 mM PIPES pH 8.0, 100X P8340), then incubated on ice for 10 min. After centrifugation at 1710 g for 5 min, pellets were resuspended in 0.5 ml ChIP SDS Lysis Buffer (1% SDS, 50 mM Tris pH 8.0, 10 mM EDTA pH 8.0, 100X P8340). The DNA was sheared by sonication on ice (10 times with 10 pulses, 50% duty, 21% output) and then precleared by incubation with 25 μl protein A/G agarose beads (Protein A/G PLUS-Agarose, Santa Cruz Biotechnology; sc-2003) for 1 hr at 4°C with rotation. To determine the DNA concentration of each sample, 50 μl of each sample was adjusted to 0.5 M NaCl, then treated with 0.3 μg/μl RNase A (Thermo Scientific; Cat no: R1253) for 30 min at 37°C, followed by treatment with 0.3 μg/μL Proteinase K (Thermo Scientific; Cat no: FEREO0492) for 2 hrs at 65°C. The DNA was then isolated using the Qiagen PCR Purification Kit (cat# 28104) according to the manufacturer’s protocol and the DNA concentration was determined by NanoDrop (Thermo Scientific). This concentration was used to calculate the lysate volume needed for input (total DNA) and IP samples. For input (total DNA) samples, sheared DNA fractions containing 240 ng of DNA (2% of IP) were diluted in 10 µl ChIP Dilution Buffer (167 mM NaCl, 16.7 mM Tris pH 8.0, 1.2 mM EDTA pH 8.0, 1.1% Triton-X 100, 0.01% SDS, 100X P8340), followed by the addition of 200 μl ChIP Elution Buffer (1% SDS, 10 mM Tris pH 8.0, 1 mM EDTA pH 8.0). For the IP samples, sheared DNA fractions containing 12 μg of DNA were diluted in 0.5 ml total ChIP Dilution Buffer combined with 2 μg BZLF1 antibody (EBV ZEBRA mouse monoclonal IgG_1_, Santa Cruz; sc-53904) or 2 μg mouse IgG_1_ isotype control (mouse (G3A1) IgG1 isotype control, Cell Signaling Technology cat# 54155), then incubated for 3 hrs at 4°C with rotation. Twenty microlitres of magnetic protein A + G beads (Magna ChIP Protein A + G Magnetic Beads, EMD Millipore Corp, cat# 16–663) were washed three times with 0.5 ml ChIP Dilution Buffer, blocked in 0.5 ml blocking buffer (0.5% BSA in PBS) for 30 min at 4°C with rotation, then added to the DNA/antibody mix for overnight incubation at 4°C with rotation. After removing the supernatant, the beads were washed three times with Low Salt Wash Buffer (150 mM NaCl, 20 mM Tris pH 8.0, 2 mM EDTA pH 8.0, 1% Triton-X 100, 0.1% SDS), then High Salt Wash Buffer (500 mM NaCl, 20 mM Tris pH 8.0, 2 mM EDTA pH 8.0, 1% Triton-X 100, 0.1% SDS), then LiCl Wash Buffer (250 mM LiCl, 10 mM Tris pH 8.0, 1 mM EDTA pH 8.0, 1% NP-40, 1% sodium deoxycholate), then with TE (10 mM Tris pH 8.0, 1 mM EDTA pH 8.0) 3 times. The supernatant was removed and immunoprecipitated complexes were eluted from the beads by incubation in 200 μl ChIP Elution Buffer at 65°C for 20 min. Cross-links were reversed from both the immunoprecipitation and total DNA samples by incubating at 65°C overnight, followed by the addition of 200 μl TE and 0.2 μg/μL RNase A. After incubation at 37°C for 2 hrs, 0.2 μg/μl Proteinase K was added followed by further incubation at 55°C for 2 hrs. The DNA was then isolated using the Qiagen PCR Purification Kit according to the manufacturer’s protocol and quantified by quantitative real-time PCR (qPCR) with the CFX384 Touch Real-Time PCR Detection System (Bio-Rad) using primers specific for IG-DMR, for MEG8 as a negative control, and for BCL2A1 as a positive control (Table 3). The percent input of chromatin recovered with the negative control IgG_1_ antibody or the BZLF1 antibody was determined for each condition. Significance was determined from three independent experiments by two-way ANOVA followed by Tukey’s multiple comparisons post-hoc test.

For ChIP experiments performed in EBV-positive cells, AGS-EBV-Z cells were seeded into two 15-cm plates per condition. Twenty-four hours later, one pair of plates was treated with 2 μg/ml doxycycline to reactivate EBV to the lytic cycle, while the other pair of plates was left untreated for the latent condition. Twenty-four hours post-reactivation, cells were cross-linked, harvested, and processed for ChIP-qPCR experiments as described above for AGS cells.

## Supporting information

S1 Fig miRNA library read depths. Read counts are shown for input lysates and Ago2-IP samples from latent (blue) and lytic (red) infections for each of the four generated libraries.
(TIF)

S1 Table Differential miRNA expression from AGS-EBV-Z cells in latent and lytic infection. Fold change of all miRNAs from input and Ago2-IP samples after lytic reactivation (compared to latent infection) are shown.
(XLSX)

S2 Table Raw miRNA counts. Raw values for miRNAs in each input sample from latent and lytic infections are shown.
(CSV)

## References

[1] Bartel DP. MicroRNAs: target recognition and regulatory functions. Cell. 2009;136(2):215–33. 19167326 10.1016/j.cell.2009.01.002 PMC3794896

[2] Pratt AJ, MacRae IJ. The RNA-induced silencing complex: a versatile gene-silencing machine. J Biol Chem. 2009;284(27):17897–901. 19342379 10.1074/jbc.R900012200 PMC2709356

[3] Friedman RC, Farh KK-H, Burge CB, Bartel DP. Most mammalian mRNAs are conserved targets of microRNAs. Genome Res. 2009;19(1):92–105. doi: 10.1101/gr.082701.108 18955434 PMC2612969

[4] Bartel DP. MicroRNAs: genomics, biogenesis, mechanism, and function. Cell. 2004;116(2):281–97. 14744438 10.1016/s0092-8674(04)00045-5

[5] Kim YK, Kim VN. Processing of intronic microRNAs. EMBO J. 2007;26(3):775–83. 17255951 10.1038/sj.emboj.7601512 PMC1794378

[6] Altuvia Y, Landgraf P, Lithwick G, Elefant N, Pfeffer S, Aravin A, et al. Clustering and conservation patterns of human microRNAs. Nucleic Acids Res. 2005;33(8):2697–706. doi: 10.1093/nar/gki567 15891114 PMC1110742

[7] Noguer-Dance M, Abu-Amero S, Al-Khtib M, Lefèvre A, Coullin P, Moore GE, et al. The primate-specific microRNA gene cluster (C19MC) is imprinted in the placenta. Hum Mol Genet. 2010;19(18):3566–82. doi: 10.1093/hmg/ddq272 20610438

[8] Bortolin-Cavaillé M-L, Dance M, Weber M, Cavaillé J. C19MC microRNAs are processed from introns of large Pol-II, non-protein-coding transcripts. Nucleic Acids Res. 2009;37(10):3464–73. doi: 10.1093/nar/gkp205 19339516 PMC2691840

[9] Morales-Prieto DM, Ospina-Prieto S, Chaiwangyen W, Schoenleben M, Markert UR. Pregnancy-associated miRNA-clusters. J Reprod Immunol. 2013;97(1):51–61. doi: 10.1016/j.jri.2012.11.001 23432872

[10] Vachon VK, Conn GL. Adenovirus VA RNA: An essential pro-viral non-coding RNA. Virus Res. 2016;212:39–52. 26116898 10.1016/j.virusres.2015.06.018

[11] Piedade D, Azevedo-Pereira JM. MicroRNAs as Important Players in Host-Adenovirus Interactions. Front Microbiol. 2017;8:1324. doi: 10.3389/fmicb.2017.01324 28769895 PMC5511817

[12] Kakavandi E, Yavarian J, Farzanehpour M, Shayestehpour M. A Review of the Interaction between miRNAs and Ebola Virus. Int J Mol Cell Med. 2024;13(2):210–9. doi: 10.22088/IJMCM.BUMS.13.2.210 39184819 PMC11344561

[13] Hull R, Marima R, Alaouna M, Demetriou D, Reis RM, Molefi T. Viral encoded miRNAs in tumorigenesis: theranostic opportunities in precision oncology. Microorganisms. 2022;10(7):1448. doi: 10.3390/microorganisms10071448 35889167 PMC9321719

[14] Dass D, Dhotre K, Chakraborty M, Nath A, Banerjee A, Bagchi P. miRNAs in herpesvirus infection: powerful regulators in small packages. Viruses. 2023;15(2):429. 36851643 10.3390/v15020429 PMC9965283

[15] Pfeffer S, Zavolan M, Grässer FA, Chien M, Russo JJ, Ju J, et al. Identification of virus-encoded microRNAs. Science. 2004;304(5671):734–6. doi: 10.1126/science.1096781 15118162

[16] Amoroso R, Fitzsimmons L, Thomas WA, Kelly GL, Rowe M, Bell AI. Quantitative studies of Epstein-Barr virus-encoded microRNAs provide novel insights into their regulation. J Virol. 2011;85(2):996–1010. doi: 10.1128/JVI.01528-10 21068248 PMC3020024

[17] Jung Y-J, Choi H, Kim H, Lee SK. MicroRNA miR-BART20-5p stabilizes Epstein-Barr virus latency by directly targeting BZLF1 and BRLF1. J Virol. 2014;88(16):9027–37. doi: 10.1128/JVI.00721-14 24899173 PMC4136301

[18] Fachko DN, Chen Y, Skalsky RL. Epstein-Barr Virus miR-BHRF1-3 Targets the BZLF1 3’UTR and Regulates the Lytic Cycle. J Virol. 2022;96(4):e0149521. doi: 10.1128/JVI.01495-21 PMC8865410 34878852

[19] Gao W, Li ZH, Chen S, Chan JYW, Yin M, Zhang MJ. Epstein-Barr virus encoded microRNA BART7 regulates radiation sensitivity of nasopharyngeal carcinoma. Oncotarget. 2017;8(12):20297–308. 28423621 10.18632/oncotarget.15526 PMC5386763

[20] Chen Y, Fachko D, Ivanov NS, Skinner CM, Skalsky RL. Epstein-Barr virus microRNAs regulate B cell receptor signal transduction and lytic reactivation. PLoS Pathog. 2019;15(1):e1007535. doi: 10.1371/journal.ppat.1007535 30615681 PMC6336353

[21] Stern-Ginossar N, Elefant N, Zimmermann A, Wolf DG, Saleh N, Biton M. Host immune system gene targeting by a viral miRNA. Science. 2007;317(5836):376–81. 17641203 10.1126/science.1140956 PMC4283197

[22] Stern-Ginossar N, Gur C, Biton M, Horwitz E, Elboim M, Stanietsky N. Human microRNAs regulate stress-induced immune responses mediated by the receptor NKG2D. Nat Immunol. 2008;9(9):1065–73. 18677316 10.1038/ni.1642

[23] Nachmani D, Stern-Ginossar N, Sarid R, Mandelboim O. Diverse herpesvirus microRNAs target the stress-induced immune ligand MICB to escape recognition by natural killer cells. Cell Host Microbe. 2009;5(4):376–85. 19380116 10.1016/j.chom.2009.03.003

[24] Choy EY-W, Siu K-L, Kok K-H, Lung RW-M, Tsang CM, To K-F, et al. An Epstein-Barr virus-encoded microRNA targets PUMA to promote host cell survival. J Exp Med. 2008;205(11):2551–60. doi: 10.1084/jem.20072581 18838543 PMC2571930

[25] Marquitz AR, Mathur A, Nam CS, Raab-Traub N. The Epstein-Barr Virus BART microRNAs target the pro-apoptotic protein Bim. Virology. 2011;412(2):392–400. doi: 10.1016/j.virol.2011.01.028 21333317 PMC3340891

[26] Harold C, Cox D, Riley KJ. Epstein-Barr viral microRNAs target caspase 3. Virol J. 2016;13(1):145. 27565721 10.1186/s12985-016-0602-7 PMC5002152

[27] Kim H, Choi H, Lee SK. Epstein-Barr virus microRNA miR-BART20-5p suppresses lytic induction by inhibiting BAD-mediated caspase-3-dependent apoptosis. J Virol. 2016;90(3):1359–68. 26581978 10.1128/JVI.02794-15 PMC4719597

[28] Zhang J, Huang T, Zhou Y, Cheng ASL, Yu J, To KF, et al. The oncogenic role of Epstein-Barr virus-encoded microRNAs in Epstein-Barr virus-associated gastric carcinoma. J Cell Mol Med. 2018;22(1):38–45. doi: 10.1111/jcmm.13354 28990284 PMC5742672

[29] Wood CD, Carvell T, Gunnell A, Ojeniyi OO, Osborne C, West MJ. Enhancer control of microRNA miR-155 expression in Epstein-Barr virus-infected B cells. J Virol. 2018;92(19):e00716-18. doi: 10.1128/JVI.00716-18 PMC6146817 30021904

[30] Kraus RJ, Perrigoue JG, Mertz JE. ZEB negatively regulates the lytic-switch BZLF1 gene promoter of Epstein-Barr virus. J Virol. 2003;77(1):199–207. doi: 10.1128/jvi.77.1.199-207.2003 12477825 PMC140584

[31] Ellis AL, Wang Z, Yu X, Mertz JE. Either ZEB1 or ZEB2/SIP1 can play a central role in regulating the Epstein-Barr virus latent-lytic switch in a cell-type-specific manner. J Virol. 2010;84(12):6139–52. doi: 10.1128/JVI.02706-09 20375168 PMC2876653

[32] Yu X, Wang Z, Mertz JE. ZEB1 regulates the latent-lytic switch in infection by Epstein-Barr virus. PLoS Pathog. 2007;3(12):e194. doi: 10.1371/journal.ppat.0030194 18085824 PMC2134958

[33] Ellis-Connell AL, Iempridee T, Xu I, Mertz JE. Cellular microRNAs 200b and 429 regulate the Epstein-Barr virus switch between latency and lytic replication. J Virol. 2010;84(19):10329–43. doi: 10.1128/JVI.00923-10 20668090 PMC2937814

[34] Lin Z, Wang X, Fewell C, Cameron J, Yin Q, Flemington EK. Differential expression of the miR-200 family microRNAs in epithelial and B cells and regulation of Epstein-Barr virus reactivation by the miR-200 family member miR-429. J Virol. 2010;84(15):7892–7. doi: 10.1128/JVI.00379-10 20484493 PMC2897641

[35] Cramer EM, Shao Y, Wang Y, Yuan Y. miR-190 is upregulated in Epstein-Barr Virus type I latency and modulates cellular mRNAs involved in cell survival and viral reactivation. Virology. 2014;464–465:184–95. 10.1016/j.virol.2014.06.029 25086243

[36] Mansouri S, Pan Q, Blencowe BJ, Claycomb JM, Frappier L. Epstein-Barr virus EBNA1 protein regulates viral latency through effects on let-7 microRNA and dicer. J Virol. 2014;88(19):11166–77. doi: 10.1128/JVI.01785-14 25031339 PMC4178782

[37] Ayoubian H, Ludwig N, Fehlmann T, Menegatti J, Gröger L, Anastasiadou E. Epstein-Barr Virus Infection of Cell Lines Derived from Diffuse Large B-Cell Lymphomas Alters MicroRNA Loading of the Ago2 Complex. J Virol. 2019;93(3):e01297-18. doi: 10.1128/JVI.01297-18 PMC6340033 30429351

[38] Dölken L, Malterer G, Erhard F, Kothe S, Friedel CC, Suffert G. Systematic analysis of viral and cellular microRNA targets in cells latently infected with human gamma-herpesviruses by RISC immunoprecipitation assay. Cell Host Microbe. 2010;7(4):324–34. 20413099 10.1016/j.chom.2010.03.008

[39] Malterer G, Dölken L, Haas J. The miRNA-targetome of KSHV and EBV in human B-cells. RNA Biol. 2011;8(1):30–4. doi: 10.4161/rna.8.1.13745 21301209

[40] Marquitz AR, Mathur A, Chugh PE, Dittmer DP, Raab-Traub N. Expression profile of microRNAs in Epstein-Barr virus-infected AGS gastric carcinoma cells. J Virol. 2014;88(2):1389–93. doi: 10.1128/JVI.02662-13 24227849 PMC3911632

[41] Oduor CI, Kaymaz Y, Chelimo K, Otieno JA, Ong’echa JM, Moormann AM, et al. Integrative microRNA and mRNA deep-sequencing expression profiling in endemic Burkitt lymphoma. BMC Cancer. 2017;17(1):761. doi: 10.1186/s12885-017-3711-9 29132323 PMC5683570

[42] Moss LI, Tompkins VS, Moss WN. Differential expression analysis comparing EBV uninfected to infected human cell lines identifies induced non-micro small non-coding RNAs. Noncoding RNA Res. 2020;5(1):32–6. doi: 10.1016/j.ncrna.2020.02.002 32154466 PMC7052066

[43] Thakur A, Kumar M. Integration of Human and Viral miRNAs in Epstein-Barr Virus-Associated Tumors and Implications for Drug Repurposing. OMICS. 2023;27(3):93–108. doi: 10.1089/omi.2023.0005 36927073

[44] De La Cruz-Herrera CF, Tatham MH, Siddiqi UZ, Shire K, Marcon E, Greenblatt JF, et al. Changes in SUMO-modified proteins in Epstein-Barr virus infection identifies reciprocal regulation of TRIM24/28/33 complexes and the lytic switch BZLF1. PLoS Pathog. 2023;19(7):e1011477. doi: 10.1371/journal.ppat.1011477 37410772 PMC10353822

[45] Lin W, Yip YL, Jia L, Deng W, Zheng H, Dai W, et al. Establishment and characterization of new tumor xenografts and cancer cell lines from EBV-positive nasopharyngeal carcinoma. Nat Commun. 2018;9(1):4663. doi: 10.1038/s41467-018-06889-5 30405107 PMC6220246

[46] Gao X, Liu H, Wang R, Huang M, Wu Q, Wang Y. Hsa-let-7d-5p promotes gastric cancer progression by targeting PRDM5. J Oncol. 2022;2022:2700651. 35847370 10.1155/2022/2700651 PMC9283079

[47] Oh ST, Seo JS, Moon UY, Kang KH, Shin DJ, Yoon SK, et al. A naturally derived gastric cancer cell line shows latency I Epstein-Barr virus infection closely resembling EBV-associated gastric cancer. Virology. 2004;320(2):330–6. 15016554 10.1016/j.virol.2003.12.005

[48] Becker-Greene D, Li H, Perez-Cremades D, Wu W, Bestepe F, Ozdemir D, et al. MiR-409-3p targets a MAP4K3-ZEB1-PLGF signaling axis and controls brown adipose tissue angiogenesis and insulin resistance. Cell Mol Life Sci. 2021;78(23):7663–79. doi: 10.1007/s00018-021-03960-1 34698882 PMC8655847

[49] Ma Z, Li Y, Xu J, Ren Q, Yao J, Tian X. MicroRNA-409-3p regulates cell invasion and metastasis by targeting ZEB1 in breast cancer. IUBMB Life. 2016;68(5):394–402. doi: 10.1002/iub.1494 27079864

[50] Wu L, Zhang Y, Huang Z, Gu H, Zhou K, Yin X, et al. MiR-409-3p inhibits cell proliferation and invasion of osteosarcoma by targeting zinc-finger e-box-binding homeobox-1. Front Pharmacol. 2019;10:137. 30846940 10.3389/fphar.2019.00137 PMC6393378

[51] Feng W, Kraus RJ, Dickerson SJ, Lim HJ, Jones RJ, Yu X, et al. ZEB1 and c-Jun levels contribute to the establishment of highly lytic Epstein-Barr virus infection in gastric AGS cells. J Virol. 2007;81(18):10113–22. doi: 10.1128/JVI.00692-07 17626078 PMC2045427

[52] McGeary SE, Lin KS, Shi CY, Pham TM, Bisaria N, Kelley GM. The biochemical basis of microRNA targeting efficacy. Science. 2019;366(6472):eaav1741. 10.1126/science.aav1741 PMC7051167 31806698

[53] González-Vallinas M, Rodríguez-Paredes M, Albrecht M, Sticht C, Stichel D, Gutekunst J, et al. Epigenetically Regulated Chromosome 14q32 miRNA Cluster Induces Metastasis and Predicts Poor Prognosis in Lung Adenocarcinoma Patients. Mol Cancer Res. 2018;16(3):390–402. doi: 10.1158/1541-7786.MCR-17-0334 29330288

[54] Murata T, Sugimoto A, Inagaki T, Yanagi Y, Watanabe T, Sato Y. Molecular basis of Epstein-Barr virus latency establishment and lytic reactivation. Viruses. 2021;13(12):2344. 34960613 10.3390/v13122344 PMC8706188

[55] Giot JF, Mikaelian I, Buisson M, Manet E, Joab I, Nicolas JC. Transcriptional interference between the EBV transcription factors EB1 and R: both DNA-binding and activation domains of EB1 are required. Nucleic Acids Research. 1991;19(6). 10.1093/nar/19.6.1251 PMC333850 1851554

[56] Zhao M, Nanbo A, Becnel D, Qin Z, Morris GF, Li L. Ubiquitin Modification of the Epstein-Barr Virus Immediate Early Transactivator Zta. J Virol. 2020;94(22):e01298-20. doi: 10.1128/JVI.01298-20 PMC7592219 32847852

[57] Flemington EK, Borras AM, Lytle JP, Speck SH. Characterization of the Epstein-Barr virus BZLF1 protein transactivation domain. J Virol. 1992;66(2):922–9. doi: 10.1128/JVI.66.2.922-929.1992 1309920 PMC240793

[58] Heston L, El-Guindy A, Countryman J, Dela Cruz C, Delecluse H-J, Miller G. Amino acids in the basic domain of Epstein-Barr virus ZEBRA protein play distinct roles in DNA binding, activation of early lytic gene expression, and promotion of viral DNA replication. J Virol. 2006;80(18):9115–33. doi: 10.1128/JVI.00909-06 16940523 PMC1563939

[59] Ramasubramanyan S, Osborn K, Al-Mohammad R, Naranjo Perez-Fernandez IB, Zuo J, Balan N, et al. Epstein-Barr virus transcription factor Zta acts through distal regulatory elements to directly control cellular gene expression. Nucleic Acids Res. 2015;43(7):3563–77. doi: 10.1093/nar/gkv212 25779048 PMC4402532

[60] Iizasa H, Wulff B-E, Alla NR, Maragkakis M, Megraw M, Hatzigeorgiou A, et al. Editing of Epstein-Barr virus-encoded BART6 microRNAs controls their dicer targeting and consequently affects viral latency. J Biol Chem. 2010;285(43):33358–70. doi: 10.1074/jbc.M110.138362 20716523 PMC2963350

[61] Qiu J, Thorley-Lawson DA. EBV microRNA BART 18-5p targets MAP3K2 to facilitate persistence in vivo by inhibiting viral replication in B cells. Proc Natl Acad Sci U S A. 2014;111(30):11157–62. 25012295 10.1073/pnas.1406136111 PMC4121837

[62] Chen Y, Fachko DN, Ivanov NS, Skalsky RL. B cell receptor-responsive miR-141 enhances Epstein-Barr virus lytic cycle via FOXO3 inhibition. mSphere. 2021;6(2):e00093-21. doi: 10.1128/mSphere.00093-21 PMC8546685 33853871

[63] Cai L, Long Y, Chong T, Cai W, Tsang CM, Zhou X, et al. EBV-miR-BART7-3p Imposes Stemness in Nasopharyngeal Carcinoma Cells by Suppressing SMAD7. Front Genet. 2019;10:939. doi: 10.3389/fgene.2019.00939 31681406 PMC6811651

[64] Shinozaki-Ushiku A, Kunita A, Isogai M, Hibiya T, Ushiku T, Takada K, et al. Profiling of Virus-Encoded MicroRNAs in Epstein-Barr Virus-Associated Gastric Carcinoma and Their Roles in Gastric Carcinogenesis. J Virol. 2015;89(10):5581–91. doi: 10.1128/JVI.03639-14 25740983 PMC4442544

[65] Imig J, Motsch N, Zhu JY, Barth S, Okoniewski M, Reineke T, et al. microRNA profiling in Epstein-Barr virus-associated B-cell lymphoma. Nucleic Acids Res. 2011;39(5):1880–93. doi: 10.1093/nar/gkq1043 21062812 PMC3061055

[66] Motsch N, Alles J, Imig J, Zhu J, Barth S, Reineke T, et al. MicroRNA profiling of Epstein-Barr virus-associated NK/T-cell lymphomas by deep sequencing. PLoS One. 2012;7(8):e42193. doi: 10.1371/journal.pone.0042193 22870299 PMC3411711

[67] Cai L-M, Lyu X-M, Luo W-R, Cui X-F, Ye Y-F, Yuan C-C, et al. EBV-miR-BART7-3p promotes the EMT and metastasis of nasopharyngeal carcinoma cells by suppressing the tumor suppressor PTEN. Oncogene. 2015;34(17):2156–66. doi: 10.1038/onc.2014.341 25347742

[68] Komabayashi Y, Kishibe K, Nagato T, Ueda S, Takahara M, Harabuchi Y. Circulating Epstein-Barr virus-encoded micro-RNAs as potential biomarkers for nasal natural killer/T-cell lymphoma. Hematol Oncol. 2017;35(4):655–63. doi: 10.1002/hon.2360 27709652

[69] Lu T, Guo Q, Lin K, Chen H, Chen Y, Xu Y, et al. Circulating Epstein-Barr virus microRNAs BART7-3p and BART13-3p as novel biomarkers in nasopharyngeal carcinoma. Cancer Sci. 2020;111(5):1711–23. doi: 10.1111/cas.14381 32155300 PMC7226202

[70] Sarshari B, Ravanshad M, Rabbani A, Zareh-Khoshchehreh R, Mokhtari F, Khanabadi B. Quantitative analysis of Epstein-Barr virus DNA in plasma and stomach biopsies of patients with gastric cancer. Virus Genes. 2023;59(3):351–8. 36757510 10.1007/s11262-023-01977-1

[71] Cai X, Schäfer A, Lu S, Bilello JP, Desrosiers RC, Edwards R. Epstein-Barr virus microRNAs are evolutionarily conserved and differentially expressed. PLoS Pathog. 2006;2(3):e23. 10.1371/journal.ppat.0020023 PMC1409806 16557291

[72] Godshalk S, Bhaduri-McIntosh S, Slack FJ. Epstein-Barr virus-mediated dysregulation of human microRNA expression. Cell Cycle. 2008;7(22):3595–600. 19001862 10.4161/cc.7.22.7120

[73] Liu Y, Li L, Shang P, Song X. LncRNA MEG8 promotes tumor progression of non-small cell lung cancer via regulating miR-107/CDK6 axis. Anticancer Drugs. 2020;31(10):1065–73. doi: 10.1097/CAD.0000000000000970 32649368

[74] Jiang M, Dai J, Jiang C, Pan Y, Ren M, Xing M. Long noncoding RNA MEG8 induces an imbalance of Th17/Treg cells through the miR-107/STAT3 axis in Henoch-Schonlein purpura rats. Aging (Albany NY). 2023;15(23):13854–64. 38054824 10.18632/aging.205266 PMC10756103

[75] Lee SM, Avalos CL, Miliotis C, Doh HM, Chan E, Kaye KM, et al. Host microRNA-31-5p represses oncogenic herpesvirus lytic reactivation by restricting the RNA-binding protein KHDRBS3-mediated viral gene expression. bioRxiv. 2025. doi: 10.1101/2025.01.22.634336

[76] Welten SMJ, Bastiaansen AJNM, De Jong RCM, De Vries MR, Peters EAB, Boonstra MC. Inhibition of 14q32 microRNAs miR-329, miR-487b, miR-494, and miR-495 increases neovascularization and blood flow recovery after ischemia. Circulation Research. 2014;115(8):696–708. 25085941 10.1161/CIRCRESAHA.114.304747

[77] Malnou EC, Umlauf D, Mouysset M, Cavaillé J. Imprinted MicroRNA Gene Clusters in the Evolution, Development, and Functions of Mammalian Placenta. Front Genet. 2019;9:706. doi: 10.3389/fgene.2018.00706 30713549 PMC6346411

[78] Enterina JR, Enfield KSS, Anderson C, Marshall EA, Ng KW, Lam WL. DLK1-DIO3 imprinted locus deregulation in development, respiratory disease, and cancer. Expert Rev Respir Med. 2017;11(9):749–61. doi: 10.1080/17476348.2017.1355241 28715922

[79] da Rocha ST, Edwards CA, Ito M, Ogata T, Ferguson-Smith AC. Genomic imprinting at the mammalian Dlk1-Dio3 domain. Trends Genet. 2008;24(6):306–16. doi: 10.1016/j.tig.2008.03.011 18471925

[80] Howard M, Charalambous M. Molecular basis of imprinting disorders affecting chromosome 14: lessons from murine models. Reproduction. 2015;149(5):R237-249. 10.1530/REP-14-0660 25820903

[81] Takada S, Tevendale M, Baker J, Georgiades P, Campbell E, Freeman T, et al. Delta-like and gtl2 are reciprocally expressed, differentially methylated linked imprinted genes on mouse chromosome 12. Curr Biol. 2000;10(18):1135–8. doi: 10.1016/s0960-9822(00)00704-1 10996796

[82] Miyoshi N, Wagatsuma H, Wakana S, Shiroishi T, Nomura M, Aisaka K, et al. Identification of an imprinted gene, Meg3/Gtl2 and its human homologue MEG3, first mapped on mouse distal chromosome 12 and human chromosome 14q. Genes Cells. 2000;5(3):211–20. doi: 10.1046/j.1365-2443.2000.00320.x 10759892

[83] Tierling S, Gasparoni G, Youngson N, Paulsen M. The Begain gene marks the centromeric boundary of the imprinted region on mouse chromosome 12. Mamm Genome. 2009;20(9–10):699–710. doi: 10.1007/s00335-009-9205-6 19641963

[84] Lin S-P, Youngson N, Takada S, Seitz H, Reik W, Paulsen M, et al. Asymmetric regulation of imprinting on the maternal and paternal chromosomes at the Dlk1-Gtl2 imprinted cluster on mouse chromosome 12. Nat Genet. 2003;35(1):97–102. doi: 10.1038/ng1233 12937418

[85] da Rocha ST, Edwards CA, Ito M, Ogata T, Ferguson-Smith AC. Genomic imprinting at the mammalian Dlk1-Dio3 domain. Trends Genet. 2008;24(6):306–16. doi: 10.1016/j.tig.2008.03.011 18471925

[86] Zeng T-B, He H-J, Han Z-B, Zhang F-W, Huang Z-J, Liu Q, et al. DNA methylation dynamics of a maternally methylated DMR in the mouse Dlk1-Dio3 domain. FEBS Lett. 2014;588(24):4665–71. doi: 10.1016/j.febslet.2014.10.038 25447521

[87] Seitz H, Royo H, Bortolin M-L, Lin S-P, Ferguson-Smith AC, Cavaillé J. A large imprinted microRNA gene cluster at the mouse Dlk1-Gtl2 domain. Genome Res. 2004;14(9):1741–8. doi: 10.1101/gr.2743304 15310658 PMC515320

[88] Baulina N, Osmak G, Kiselev I, Popova E, Boyko A, Kulakova O, et al. MiRNAs from DLK1-DIO3 Imprinted Locus at 14q32 are Associated with Multiple Sclerosis: Gender-Specific Expression and Regulation of Receptor Tyrosine Kinases Signaling. Cells. 2019;8(2):133. doi: 10.3390/cells8020133 30743997 PMC6406543

[89] Srinath S, Jishnu PV, Varghese VK, Shukla V, Adiga D, Mallya S, et al. Regulation and tumor-suppressive function of the miR-379/miR-656 (C14MC) cluster in cervical cancer. Mol Oncol. 2024;18(6):1608–30. doi: 10.1002/1878-0261.13611 38400534 PMC11161731

[90] Valdmanis PN, Roy-Chaudhuri B, Kim HK, Sayles LC, Zheng Y, Chuang CH. Upregulation of the microRNA cluster at the Dlk1-Dio3 locus in lung adenocarcinoma. Oncogene. 2015;34(1):94–103. 24317514 10.1038/onc.2013.523 PMC4065842

[91] Milosevic J, Pandit K, Magister M, Rabinovich E, Ellwanger DC, Yu G, et al. Profibrotic role of miR-154 in pulmonary fibrosis. Am J Respir Cell Mol Biol. 2012;47(6):879–87. doi: 10.1165/rcmb.2011-0377OC 23043088 PMC3547095

[92] Qu R, Chen X, Zhang C. LncRNA ZEB1-AS1/miR-409-3p/ZEB1 feedback loop is involved in the progression of non-small cell lung cancer. Biochem Biophys Res Commun. 2018;507(1–4):450–6. 30448056 10.1016/j.bbrc.2018.11.059

[93] Robbiani DF, Nussenzweig MC. Chromosome translocation, B cell lymphoma, and activation-induced cytidine deaminase. Annu Rev Pathol. 2013;8:79–103. 22974238 10.1146/annurev-pathol-020712-164004

[94] Katrincsakova B, Takeda H, Urbankova H, Michaux L, Jarosova M, Vandenberghe P. Methylation analysis of the imprinted DLK1-GTL2 domain supports the random parental origin of the IGH-involving del(14q) in B-cell malignancies. Epigenetics. 2009;4(7):469–75. 19786834 10.4161/epi.4.7.9924

[95] Chapman CJ, Mockridge CI, Rowe M, Rickinson AB, Stevenson FK. Analysis of VH genes used by neoplastic B cells in endemic Burkitt’s lymphoma shows somatic hypermutation and intraclonal heterogeneity. Blood. 1995;85(8):2176–81. 7718888

[96] Chen C, Li D, Guo N. Regulation of cellular and viral protein expression by the Epstein-Barr virus transcriptional regulator Zta: implications for therapy of EBV associated tumors. Cancer Biol Ther. 2009;8(11):987–95. doi: 10.4161/cbt.8.11.8369 19448399

[97] Hara S, Terao M, Tsuji-Hosokawa A, Ogawa Y, Takada S. Humanization of a tandem repeat in IG-DMR causes stochastic restoration of paternal imprinting at mouse Dlk1-Dio3 domain. Hum Mol Genet. 2021;30(7). 10.1093/hmg/ddab071 PMC8120134 33709141

[98] Lu HP, Lin CJ, Chen WC, Chang YJ, Lin SW, Wang HH. TRIM28 regulates Dlk1 expression in adipogenesis. Int J Mol Sci. 2020;21(19):7245. 33008113 10.3390/ijms21197245 PMC7582669

[99] Takahashi N, Gray D, Strogantsev R, Noon A, Delahaye C, Skarnes WC, et al. ZFP57 and the Targeted Maintenance of Postfertilization Genomic Imprints. Cold Spring Harb Symp Quant Biol. 2015;80:177–87. doi: 10.1101/sqb.2015.80.027466 27325708

[100] Alexander KA, Wang X, Shibata M, Clark AG, García-García MJ. TRIM28 Controls Genomic Imprinting through Distinct Mechanisms during and after Early Genome-wide Reprogramming. Cell Rep. 2015;13(6):1194–205. doi: 10.1016/j.celrep.2015.09.078 26527006 PMC4644443

[101] Salamun SG, Sitz J, De La Cruz-Herrera CF, Yockteng-Melgar J, Marcon E, Greenblatt J, et al. The Epstein-Barr Virus BMRF1 Protein Activates Transcription and Inhibits the DNA Damage Response by Binding NuRD. J Virol. 2019;93(22):e01070-19. 10.1128/JVI.01070-19 PMC6819917 31462557

[102] Campbell AM, De La Cruz-Herrera CF, Marcon E, Greenblatt J, Frappier L. Epstein-Barr Virus BGLF2 commandeers RISC to interfere with cellular miRNA function. PLoS Pathog. 2022;18(1):e1010235. doi: 10.1371/journal.ppat.1010235 35007297 PMC8782528

[103] Chan SY-Y, Choy K-W, Tsao S-W, Tao Q, Tang T, Chung GT-Y, et al. Authentication of nasopharyngeal carcinoma tumor lines. Int J Cancer. 2008;122(9):2169–71. doi: 10.1002/ijc.23374 18196576

[104] Strong MJ, Baddoo M, Nanbo A, Xu M, Puetter A, Lin Z. Comprehensive high-throughput RNA sequencing analysis reveals contamination of multiple nasopharyngeal carcinoma cell lines with HeLa cell genomes. J Virol. 2014;88(18):10696–704. doi: 10.1128/JVI.01457-14 24991015 PMC4178894

[105] Friedländer MR, Mackowiak SD, Li N, Chen W, Rajewsky N. miRDeep2 accurately identifies known and hundreds of novel microRNA genes in seven animal clades. Nucleic Acids Res. 2012;40(1):37–52. doi: 10.1093/nar/gkr688 21911355 PMC3245920

[106] Robinson MD, McCarthy DJ, Smyth GK. edgeR: a Bioconductor package for differential expression analysis of digital gene expression data. Bioinformatics. 2010;26(1):139–40. 19910308 10.1093/bioinformatics/btp616 PMC2796818

[107] McCarthy DJ, Chen Y, Smyth GK. Differential expression analysis of multifactor RNA-Seq experiments with respect to biological variation. Nucleic Acids Res. 2012;40(10):4288–97. doi: 10.1093/nar/gks042 22287627 PMC3378882

[108] Kozomara A, Griffiths-Jones S. miRBase: annotating high confidence microRNAs using deep sequencing data. Nucleic Acids Res. 2014;42(Database issue):D68-73. doi: 10.1093/nar/gkt1181 24275495 PMC3965103

[109] Griffiths-Jones S. The microRNA Registry. Nucleic Acids Res. 2004;32(Database issue):D109-111. 10.1093/nar/gkh023 PMC308757 14681370

[110] Griffiths-Jones S, Grocock RJ, van Dongen S, Bateman A, Enright AJ. miRBase: microRNA sequences, targets and gene nomenclature. Nucleic Acids Res. 2006;34(Database issue):D140-144. 10.1093/nar/gkj112 PMC1347474 16381832

[111] Griffiths-Jones S, Saini HK, van Dongen S, Enright AJ. miRBase: tools for microRNA genomics. Nucleic Acids Res. 2008;36(Database issue):D154-8. doi: 10.1093/nar/gkm952 17991681 PMC2238936

[112] Kozomara A, Birgaoanu M, Griffiths-Jones S. miRBase: from microRNA sequences to function. Nucleic Acids Research. 2019;47(D1):D155-62. 10.1093/nar/gky1141 PMC6323917 30423142

[113] Kagami M, Sekita Y, Nishimura G, Irie M, Kato F, Okada M, et al. Deletions and epimutations affecting the human 14q32.2 imprinted region in individuals with paternal and maternal upd(14)-like phenotypes. Nat Genet. 2008;40(2):237–42. doi: 10.1038/ng.2007.56 18176563

[114] De La Cruz-Herrera CF, Shire K, Siddiqi UZ, Frappier L. A genome-wide screen of Epstein-Barr virus proteins that modulate host SUMOylation identifies a SUMO E3 ligase conserved in herpesviruses. PLoS Pathog. 2018;14(7):e1007176. doi: 10.1371/journal.ppat.1007176 29979787 PMC6051671

[115] Wen W, Iwakiri D, Yamamoto K, Maruo S, Kanda T, Takada K. Epstein-Barr virus BZLF1 gene, a switch from latency to lytic infection, is expressed as an immediate-early gene after primary infection of B lymphocytes. J Virol. 2007;81(2):1037–42. doi: 10.1128/JVI.01416-06 17079287 PMC1797481

[116] Murata T, Sato Y, Nakayama S, Kudoh A, Iwahori S, Isomura H, et al. TORC2, a coactivator of cAMP-response element-binding protein, promotes Epstein-Barr virus reactivation from latency through interaction with viral BZLF1 protein. J Biol Chem. 2009;284(12):8033–41. doi: 10.1074/jbc.M808466200 19164291 PMC2658097

