# Supplementary figures and images for: Profiling miRNA changes in Epstein-Barr virus lytic infection identifies a function for BZLF1 in upregulating miRNAs from the DLK1-DIO3 locus
Source: PLoS Pathog. 2025 Jul 17;21(7):e1013347. doi: 10.1371/journal.ppat.1013347 (PMC12286400; doi:10.1371/journal.ppat.1013347)

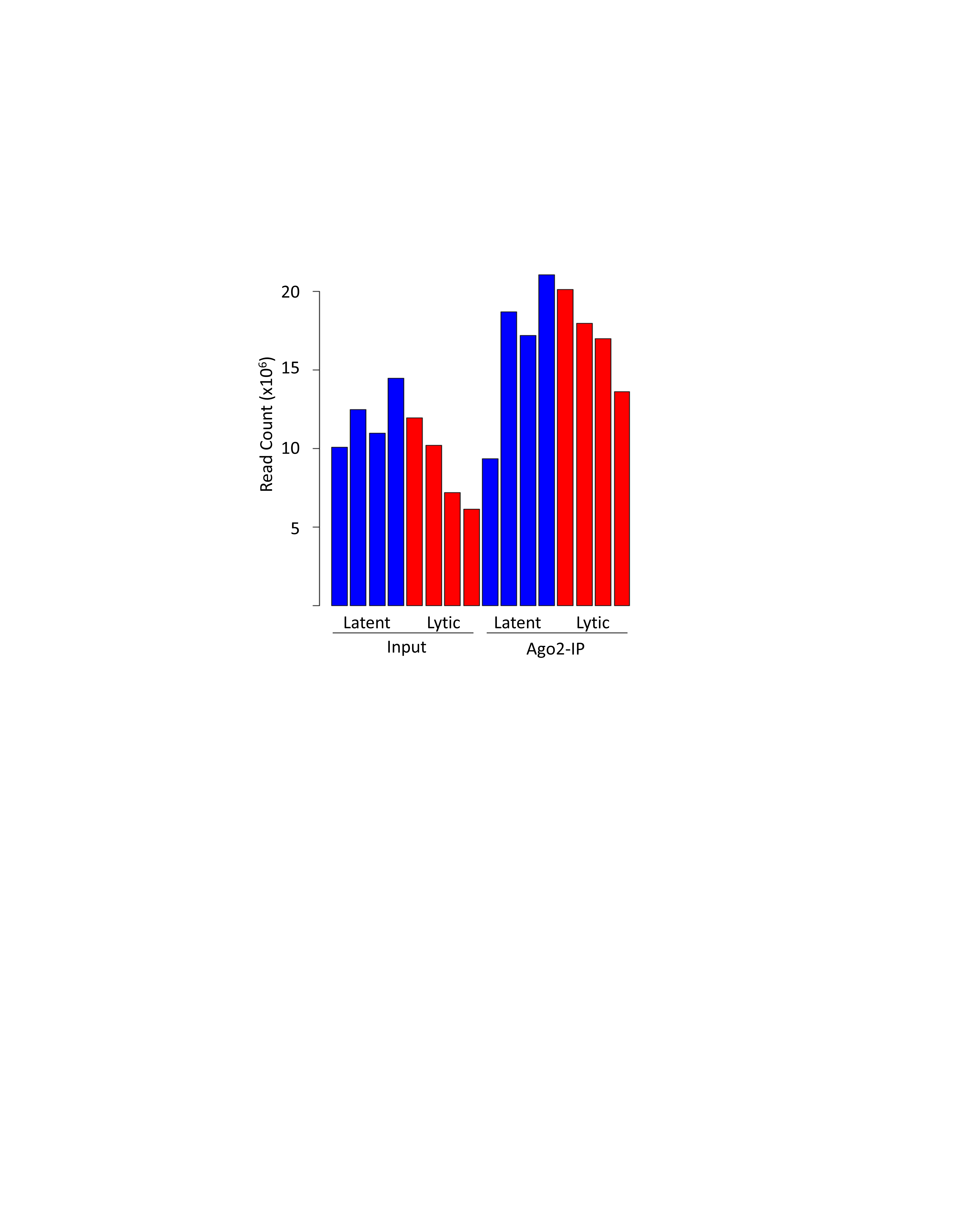

Supplement: S1 Fig — Read counts are shown for input lysates and Ago2-IP samples from latent (blue) and lytic (red) infections for each of the four generated libraries. (TIF) [file ppat.1013347.s001.tif]
